# Transition from carbonatitic magmas to hydrothermal brines: Continuous dilution or fluid exsolution?

**DOI:** 10.1126/sciadv.adh0458

**Published:** 2023-07-19

**Authors:** Xueyin Yuan, Richen Zhong, Xin Xiong, Jing Gao, Yubo Ma

**Affiliations:** ^1^ MNR Key Laboratory of Metallogeny and Mineral Assessment, Institute of Mineral Resources, Chinese Academy of Geological Sciences, Beijing 100037, China.; ^2^ School of Civil and Resource Engineering, University of Science and Technology Beijing, Beijing 100083, China.; ^3^ Laboratory of Extraterrestrial Ocean Systems, Institute of Deep-sea Science and Engineering, Chinese Academy of Sciences, Sanya 572000, China.

## Abstract

Carbonatites are the most important primary sources for the rare earth elements (REEs). While fractional crystallization of carbonate minerals results in the enrichment of volatiles, alkalis, and REEs in the remaining melts, the transition from carbonatitic magmas to hydrothermal brines remains unclear. Here, we investigated the pressure-temperature-composition (*P*-*T*-*X*) properties of the Na_2_CO_3_-H_2_O system up to 700°C and 11.0 kbar using a hydrothermal diamond anvil cell and a Raman spectrometer. Our results show that Na_2_CO_3_ becomes increasingly soluble under high *P-T* conditions, leading to the disappearance of melt-fluid immiscibility and the continuous transition from Na_2_CO_3_ melts to hydrothermal brines under deep crustal conditions. Given the abundance of Na_2_CO_3_ in highly evolved carbonatitic systems, we suggest that the continuous melt-fluid transition in deep-seated carbonatites results in REEs being sufficiently concentrated in the brine-melts to form economic ore bodies, whereas in shallow systems, REEs preferentially partition into carbonatitic magmas over synmagmatic brines and disperse in carbonatite rocks that underwent limited fractionation.

## Teaser

Distinct melt-fluid transitions in carbonatites: Continuous evolution in the deep, fluid exsolution in the shallow.

## INTRODUCTION

Carbonatites are igneous rocks containing carbonate minerals that crystallized from melts with carbonate predominating over all other anions combined (*1*). These rocks continue to receive considerable attention due to their peculiar compositions and unique enrichment in incompatible trace elements, particularly the rare earth elements (REEs), which are of strategic importance in the fields of renewable energies, catalysts, electronics, and modern defense systems (*2*, *3*). The more than 600 carbonatites worldwide are dominated by Ca-, Mg-, and Fe-carbonates (*4*, *5*), with the sole exception being the natrocarbonatite at Oldoinyo Lengai, Tanzania (*6*). However, the presence of alkali-rich melt inclusions in calciocarbonatites (*2*, *7*, *8*) and the widespread fenitization (alkaline metasomatism) of the country rocks (*1*, *9*, *10*) reveal that the parental carbonatitic magmas were rich in alkalis, with experimental evidence further supporting the crystallization of alkali-poor cumulates from alkali-rich melts (*11*–*13*). The fractional crystallization of Ca-, Mg-, and Fe-carbonates gives rise to the residual melts being increasingly enriched in alkalis, volatiles, and incompatible elements (*12*–*17*). These components act as fluxes that further depress the solidi of the carbonatitic melts, until the melts become volatile- and alkali-saturated with distinct chemical properties from their parental magmas (*1*, *12*, *14*).

The proposed melt-fluid evolution processes in carbonatitic systems include continuous transition from carbonatitic magmas into hydrothermal brines via crystallization of carbonate, phosphate, and, in some cases, sulfate and fluoride minerals (*10*, *14*, *18*), and/or exsolution of synmagmatic brines from carbonatitic magmas (degassing) (*19*, *20*). Here, we quote the term “brine-melt” from (*1*, *8*, *14*) to describe the alkali- and volatile-rich carbonatitic melts that evolved from carbonatitic magmas by fractionating calcite, dolomite, apatite, and other magmatic minerals. The melt-fluid evolution processes potentially result in distinct mineralization of REEs in carbonatites. While exsolution of synmagmatic brines occurs, both major and trace elements (Ca, Mg, Na, Sr, Ba, REEs, etc.) partition strongly into carbonatitic melts over aqueous fluids ($D_{\mathrm{REE}}^{F/M}$ = 0.02 to 0.15), with REEs crystallizing as apatite, monazite, or other magmatic minerals (*13*, *21*, *22*). The brines exsolved from carbonatitic magmas contribute to the secondary alteration and redistribution of REEs, instead of the primary mineralization of them (*23*, *24*). Whereas during continuous melt-fluid evolution, REEs will be sufficiently concentrated in the brine-melts, as evident by the presence of REE daughter minerals and high REE contents [up to 3 weight % (wt %)] in the melt-fluid inclusions (*10*, *25*–*30*). In these scenarios, REEs crystallize either as ephemeral burbankite or carbocernaite group minerals in silica-poor systems, which subsequently alter into ancylite, bastnäsite with synchysite, or parisite (*11*, *24*, *30*–*32*), or directly as bastnäsite and parisite in silica-rich systems (*11*, *18*, *24*, *33*). Nevertheless, our current knowledge about the effect of melt-fluid evolution through degassing versus crystallization, or how the presence or absence of melt-fluid immiscibility will affect the composition of carbonatitic fluids or brine-melts, remains limited.

A better understanding of the melt-fluid transition in carbonatites and its role in the development of carbonatitic REE deposits thus requires constraining the *P*-*T*-*X* properties of salt-water systems involving Na_2_CO_3_ and K_2_CO_3_. Experimental studies show that K_2_CO_3_ has a prograde solubility, which increases from 7.63 mol/kg (51.3 wt %) at 0°C to 30.85 mol/kg (81.0 wt %) at 450°C under vapor pressure and can be extrapolated to the K_2_CO_3_ melting point at 891°C (*34*, *35*). In other words, there is no fluid exsolution from K_2_CO_3_ melts, but instead, a continuous transition from K_2_CO_3_ melts to K_2_CO_3_-bearing fluids upon cooling. In contrast, the solubility of Na_2_CO_3_ under vapor pressure is retrograde, decreasing from 4.2 mol/kg (30.9 wt %) at 100°C to almost 0 around the critical point of H_2_O (*36*–*39*). With further increase in *P-T* conditions, hydrous melting of Na_2_CO_3_ occurs around 500°C and 1.5 kbar, resulting in the coexistence of a Na_2_CO_3_ melt with an aqueous fluid (melt-fluid immiscibility) under higher *P*-*T* conditions (*40*, *41*). Given the much greater abundance of Na over K in carbonatitic magmas (*2*, *7*) and associated hydrothermal brines (*10*, *29*, *42*), this marked difference between the Na_2_CO_3_-H_2_O and K_2_CO_3_-H_2_O systems hints at the exsolution of synmagmatic brines being very common in the development of carbonatitic intrusions under shallow crustal conditions. However, we also noticed that with the increase in pressure from 0.5 to 2.0 kbar, the Na_2_CO_3_ melt and aqueous fluid become increasingly miscible (*43*), implying that the melt-fluid evolution could be continuous under deep crustal conditions. Unfortunately, investigation of the thermodynamic properties of the Na_2_CO_3_-H_2_O system is currently limited to pressures below 2.0 kbar (*40*, *41*, *43*), much lower than the *P*-*T* condition (600° to 850°C, 3.0 to 15.7 kbar) for melt-fluid transition in deep-seated carbonatites (*8*, *10*, *18*, *44*, *45*). Here, to determine the transition from carbonatitic magmas to hydrothermal brines in the deep crust, we investigated the phase relations in the Na_2_CO_3_-H_2_O system at *P-T* conditions up to 700°C and 11.0 kbar, along with the influence from K_2_CO_3_ or CaCO_3_ on the melt-fluid transitions under shallow and deep crustal conditions.

## RESULTS

### Exsolution of synmagmatic brines from Na_2_CO_3_ melts under shallow conditions

**Fig. 1.**
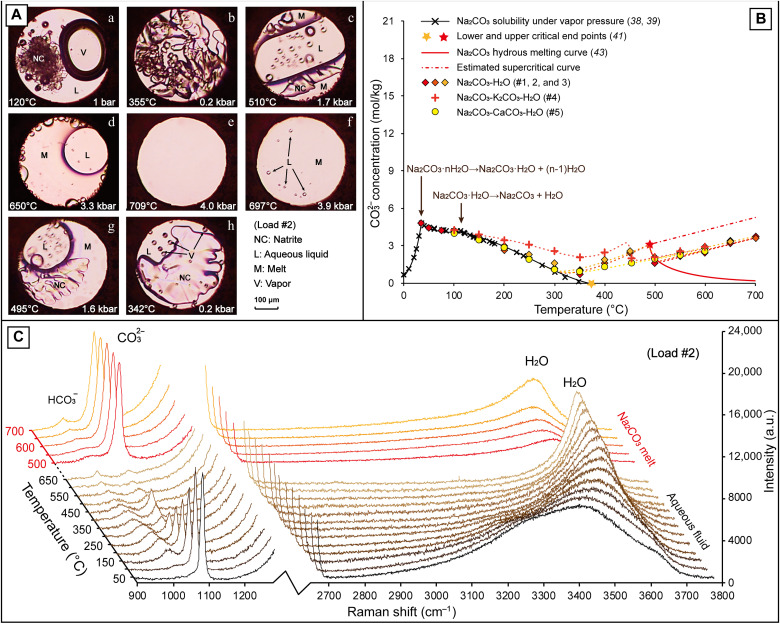
Exsolution of an aqueous brine from a Na_2_CO_3_ melt under shallow crustal conditions. (**A**) Photomicrographs showing the phase transitions in the Na_2_CO_3_-H_2_O system (#2) during heating (a to e) and cooling (f to h). (**B**) Raman quantification results for CO_3_^2−^ concentration in the aqueous fluids from loads #1 to 5, with the Na_2_CO_3_ hydrous melting curve, upper critical end point, and estimated supercritical curve from Veksler (*41*) and Koster van Groos (*43*). (**C**) Variations in CO_3_^2−^ and H_2_O Raman peak intensities in the Raman spectra measured from the aqueous fluid and Na_2_CO_3_ melt in load #2.

**Fig. 2.**
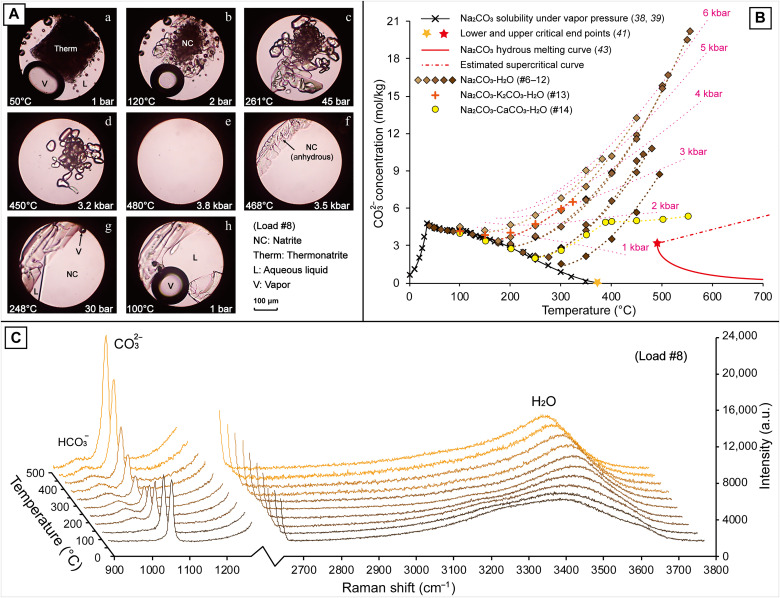
Continuous transition between an aqueous fluid and a Na_2_CO_3_ brine-melt under deep crustal conditions. (**A**) Photomicrographs showing the phase changes in the Na_2_CO_3_-H_2_O system (#8) during heating (a to e) and cooling (f to h). (**B**) Raman quantification results for CO_3_^2−^ concentration under high *P-T* conditions in loads #6 to 14, with pink dotted lines showing the isobaric curves. (**C**) Variations in CO_3_^2−^ and H_2_O Raman peak intensities with increasing temperature in load #8.

A summary of the phase changes in the Na_2_CO_3_-H_2_O system along different *P*-*T* paths is listed in Table 1. While heating the Na_2_CO_3_-H_2_O samples (loads #1 to 3 and #6 to 12) under vapor pressure (i.e., before disappearance of the vapor bubbles), the phase changes observed in all loads include dehydration from thermonatrite (Na_2_CO_3_·H_2_O) into anhydrous natrite (Na_2_CO_3_) around 110°C (Figs. 1A, a, and 2A, a and b), followed by an increase in the natrite crystal volume (Figs. 1A, a and b, and 2A, b and c) due to the retrograde Na_2_CO_3_ solubility (*39*). The decreasing Na_2_CO_3_ concentration in the fluids can also be easily noticed from the decrease in CO_3_^2−^ Raman peak intensity (Figs. 1C and 2C), with Raman quantification results showing the CO_3_^2−^ concentration decreasing from 4.20 mol/kg (30.8 wt %) at 100°C to around 1.0 mol/kg (9.6 wt %) at 300°C (Figs. 1B and 2B), which agrees well with the Na_2_CO_3_ solubility data from other high-temperature experiments (*38*, *39*).

**Table 1.** Summary of the phase transitions in the Na_2_CO_3_-H_2_O system under high *P-T* conditions.

Load #	Sample composition	Phase transitions*	Vapor homogenization	Na_2_CO_3_ melting	Na_2_CO_3_-H_2_O homogenization			
					*P*-*T* condition†	mol/kg‡	Weight %‡	ρ (g/cm^3^)§
1	Na_2_CO_3_ + H_2_O	S_1_ + L + V → S_1_ + L → BM + L	376°C, 0.3 kbar	530°C, 1.5 kbar	–¶	3.77	28.6	0.755
2	Na_2_CO_3_ + H_2_O	S_1_ + L + V → S_1_ + L → BM + L → BM	355°C, 0.2 kbar	500°C, 1.6 kbar	709°C, 4.0 kbar	13.52	58.9	1.511
3	Na_2_CO_3_ + H_2_O	S_1_ + L + V → S_1_ + L → BM + L	345°C, 0.2 kbar	500°C, 1.6 kbar	–	3.63	27.8	0.944
4	Na_2_CO_3_ + K_2_CO_3_ + H_2_O	S_1_ + L + V → S_1_ + L → BM + L	352°C, 0.2 kbar	450°C, 1.1 kbar	–	2.72	22.4	0.858
5	Na_2_CO_3_ + H_2_O + Calcite	S_1_ + S_2_ + L + V → S_1_ + S_2_ + L → S_2_ + BM + L → BM + L	323°C, 0.1 kbar	530°C, 2.5 kbar	–	2.59	21.6	0.930
6	Na_2_CO_3_ + H_2_O	S_1_ + L + V → S_1_ + L → BM	253°C, 40 bar	–	462°C, 3.7 kbar	10.37	52.4	1.512
7	Na_2_CO_3_ + H_2_O	S_1_ + L + V → S_1_ + L → BM	298°C, 80 bar	–	495°C, 2.8 kbar	8.75	48.1	1.387
8	Na_2_CO_3_ + H_2_O	S_1_ + L + V → S_1_ + L → BM	261°C, 45 bar	–	480°C, 3.8 kbar	10.81	53.4	1.532
9	Na_2_CO_3_ + H_2_O	S_1_ + L + V → S_1_ + L → BM	212°C, 20 bar	–	555°C, 6.6 kbar	20.19	68.2	1.825
10	Na_2_CO_3_ + H_2_O	S_1_ + L + V → S_1_ + L → BM	200°C, 16 bar	–	512°C, 5.8 kbar	16.66	63.9	1.751
11	Na_2_CO_3_ + H_2_O	S_1_ + L + V → S_1_ + L → BM	164°C, 5 bar	–	450°C, 5.9 kbar	13.25	58.4	1.678
12	Na_2_CO_3_ + H_2_O	S_1_ + L + V → S_1_ + L → BM	124°C, 2 bar	–	381°C, 5.2 kbar	9.98	51.4	1.584
13	Na_2_CO_3_ + K_2_CO_3_ + H_2_O	S_1_ + L + V → S_1_ + L → BM	147°C, 4 bar	–	320°C, 3.7 kbar	6.58	41.1	1.418
14	Na_2_CO_3_ + H_2_O + Calcite	S_1_ + S_2_ + L + V → S_1_ + S_2_ + L → S_2_ + BM	238°C, 33 bar	–	384°C, 2.6 kbar	4.99	34.6	1.237
15	Na_2_CO_3_ + H_2_O + Quartz	S_1_ + S_2_ + L	–	–	–	5.45	36.6	1.567
16	Na_2_CO_3_ + H_2_O + Quartz	S_1_ + S_2_ + L	–	–	–	6.13	39.4	1.572

*S_1_ is for Na_2_CO_3_ solid (thermonatrite or natrite); S_2_ is for other solids (calcite, nyerereite, or quartz); L, V, and BM denote aqueous fluid, vapor, and Na_2_CO_3_ brine-melt (including melts that coexisted with an aqueous fluid and condensed liquids with Na_2_CO_3_ centration greater than the supercritical curve), respectively.

†Pressure was estimated from isochoric *P-T* curves defined for the NaCl-H_2_O system (#1 to 4 and 6 to 13) (*63*) or calibrated using the frequency shifts in the 1086 cm^−1^ peak of calcite (#5 and 14) (*64*) or the 464 cm^−1^ peak of quartz (#15 and 16) (*61*).

‡Na_2_CO_3_ concentration in the homogeneous Na_2_CO_3_ brine-melts (#2 and 6 to 14) or in the aqueous fluids equilibrated with a Na_2_CO_3_ melt (#1 and 3 to 5) or natrite solid (#15 and 16).

§Fluid density was estimated by extrapolating the variation in density (*68*, *69*) with changes in temperature and Na_2_CO_3_ concentration.

¶“–” for not exist or not observed.

In the three Na_2_CO_3_-H_2_O loads (#1 to 3) with fluid pressure below 1.0 kbar at 400°C, hydrous melting of natrite occurred between 500° and 530°C under 1.6 kbar (Fig. 1A, c), consistent with the Na_2_CO_3_ hydrous melting curve reported by van Groos (*43*). Subsequently, coexistence between a Na_2_CO_3_ melt and an aqueous fluid continued up to 700°C and 4.0 kbar, with progressive assimilation of the aqueous fluid by the Na_2_CO_3_ melt (Fig. 1A, d), until the system homogenized into a hydrous melt phase (brine-melt) under 709°C and 4.0 kbar in load #2 (Fig. 1A, e, melt-fluid homogenization in the other two loads was estimated to be >750°C). Upon cooling of the homogeneous brine-melt in load #2, aqueous fluid droplets emerged at 697°C (Fig. 1A, f), then melt-fluid coexisted, and the melt crystallized into natrite around 490°C (Fig. 1A, g). Thereafter, separation of vapor bubbles from the fluid occurred at 343°C (Fig. 1A, h), followed by partial dissolution of the natrite crystals with a further decrease in temperature, indicating that the phase changes in the Na_2_CO_3_-H_2_O system were reversible.

The CO_3_^2−^ Raman peak intensity of the aqueous fluids in loads #1 to 3 increased slightly in the 350° to 500°C temperature range, then dropped again upon melting of natrite around 500°C, and remained very low within the melt-fluid immiscibility field (Fig. 1C). In the meantime, the O-H peak intensity of the Na_2_CO_3_ melt increased noticeably with increasing temperature (Fig. 1C), implying that the Na_2_CO_3_ melt became increasingly hydrous under higher *P-T* conditions. Unfortunately, Raman quantification for the fluid and melt compositions in these loads (#1 to 3) were considerably inaccurate, due to the very weak CO_3_^2−^ Raman signal in the aqueous fluids or the weak and wide O-H band in the Na_2_CO_3_ melts (Fig. 1C), which complicated the peak fitting procedures and resulted in larger uncertainties (*46*). Moreover, a weak HCO_3_^−^ peak was detected in these Raman spectra (Fig. 1C), implying that a large proportion (>60%) of CO_3_^2−^ transformed into HCO_3_^−^ in the aqueous fluids, which potentially resulted in an overestimation of the CO_3_^2−^ concentration with our calculation method (see the Supplementary Materials). Nevertheless, by comparing the Na_2_CO_3_ concentration in the homogeneous melt in load #2 (13.52 mol/kg or 58.9 wt %) and the aqueous fluids that coexisted with a melt in loads #1 to 3 (1.97 to 3.77 mol/kg or 17.3 to 28.6 wt %; table S1), the fluid-melt distribution coefficient of Na ($D_{\mathrm{Na}}^{F/M}$) was estimated to be <0.25, consistent with the results (0.10 to 0.35) from other high *P-T* experiments (*21*, *22*).

The addition of K_2_CO_3_ (load #4) or calcite (CaCO_3_, load #5) to the Na_2_CO_3_-H_2_O system did not preclude the occurrence of melt-fluid immiscibility under shallow crustal conditions. However, while Na_2_CO_3_ was loaded with K_2_CO_3_ solution (1.0 mol/kg) in load #4, the CO_3_^2−^ concentration between 200° and 400°C was higher than that in the Na_2_CO_3_-H_2_O system (load #2, with a similar *P-T* path) by 0.42 to 1.08 mol/kg; meanwhile, the natrite melting temperature also decreased by about 50°C. The addition of calcite in load #5 did not cause detectable variations in fluid composition at *T* ≤ 350°C, whereas under higher *P-T* conditions, the formation of nyerereite [Na_2_Ca(CO_3_)_2_] from reaction between Na_2_CO_3_ and calcite (fig. S1) resulted in the CO_3_^2−^ concentration decreasing by 0.26 to 0.79 mol/kg between 400° and 500°C. Regardless of the uncertainties in Raman quantification results, the fluid compositions in loads #1 to 5 were consistent at *T* > 500°C (Fig. 1B), indicating that the synmagmatic brines exsolved from carbonatitic magmas are consistently characterized by low CO_3_^2−^ concentrations (e.g., <4.0 mol/kg or 30 wt %).

### Properties of carbonatitic brine-melts under deep crustal conditions

In the seven Na_2_CO_3_-H_2_O loads (#6 to 12) with fluid pressure greater than 1.0 kbar at 400°C, the natrite crystals were observed to dissolve progressively into the liquids at temperatures exceeding liquid-vapor homogenization (Fig. 2A, c and d), until the samples homogenized (Fig. 2A, e) at *P-T* conditions ranging from 381° to 555°C and between 2.8 and 6.6 kbar (Table 1). The CO_3_^2−^ Raman peak intensity increased accordingly (Fig. 2C), showing the transition of Na_2_CO_3_ solubility from retrograde to intensely prograde with increasing pressure. The CO_3_^2−^ concentration in the homogeneous liquids ranged between 8.75 and 20.19 mol/kg (48.1 to 68.2 wt %), comparable to the brine-melt obtained in load #2 (13.52 mol/kg or 58.9 wt %), and are much greater than the supercritical liquid at the upper critical end point (around 4.0 mol/kg or 30 wt %; Fig. 2B) of the Na_2_CO_3_-H_2_O system (*40*, *41*). These results imply that the homogeneous liquids in loads #6 to 12 were hydrous Na_2_CO_3_ melts or brine-melts, resulting from miscibility between aqueous fluids and Na_2_CO_3_ melts. Upon cooling, natrite crystals precipitated from the brine-melts at 10° to 20°C below the homogenization temperature (Fig. 2A, f). Thereafter, the crystals grew quickly along with the decrease in CO_3_^2−^ concentration, showing the continuous transition from brine-melts into hydrothermal brines via crystallization of carbonate minerals. With the further decease in temperature, vapor bubbles appeared at 10° to 20°C below the liquid-vapor homogenization (Fig. 2A, g), followed by partial dissolution of the natrite crystals into the fluids again (Fig. 2A, h).

The addition of K_2_CO_3_ solution (1 mol/kg; load #13) into the high-pressure Na_2_CO_3_-H_2_O system did not cause obvious variation in CO_3_^2−^ concentration (Fig. 2B). However, while calcite was added to the system (load #14), the formation of nyerereite at *T* > 300°C (fig. S2) suppressed the increase in CO_3_^2−^ concentration with increasing *P-T* condition. In contrast to the CO_3_^2−^ concentration in load #6, which increased from 2.86 mol/kg (23.3 wt %) at 300°C and 0.7 kbar to 6.59 mol/kg (41.1 wt %) at 400°C and 2.5 kbar, that in load #14 was lower by 0.53 mol/kg at 350°C and 2.0 kbar, and by 0.98 mol/kg at 384°C and 2.6 kbar (table S1). With the further increase in *P-T* condition, nyerereite dissolved under 550°C and 5.6 kbar, which resulted in a slight increase in CO_3_^2−^ concentration by another 0.33 mol/kg (Fig. 2B). In summary, the phase transition from carbonatitic brine-melts to hydrothermal brines under deep crustal conditions is continuous with no clear phase boundary, which is distinct from that under shallow conditions, but instead similar to the K_2_CO_3_-H_2_O system (*40*, *41*).

### *P*-*T*-*X* phase relations of the Na_2_CO_3_-H_2_O system

**Fig. 3.**
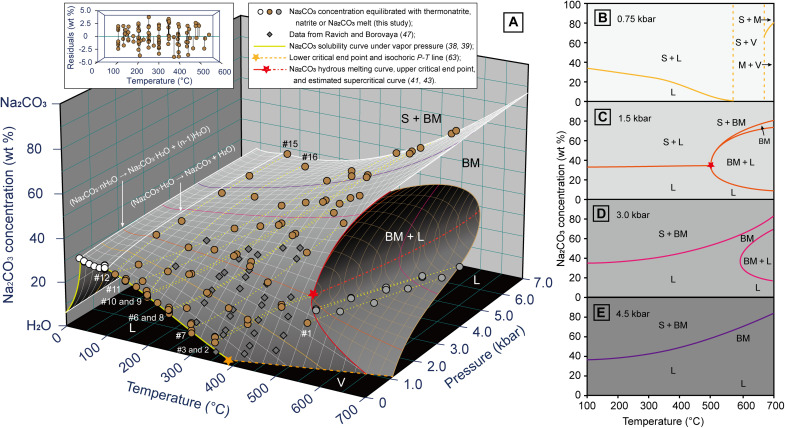
*P*-*T*-*X* phase diagram of the Na_2_CO_3_-H_2_O system. (**A**) A 3D diagram showing the Na_2_CO_3_ saturation concentration and melt-fluid immiscibility zone under high *P-T* conditions; the Na_2_CO_3_ concentration equilibrated with natrite (brown circles) was fitted as a third-order polynomial function of pressure and temperature, with residuals being shown in the inset. (**B** to **E**) Isobaric sections of the *P-T-X* diagram at 0.75, 1.5, 3.0 and 4.5 kbar, respectively. S, M, BM, L, and V denote natrite solid, Na_2_CO_3_ melt, brine-melt, aqueous liquid, and vapor, respectively. See the main text for descriptions of the phase relations in the Na_2_CO_3_-H_2_O system.

By combining the results from our present experiment (table S1) and those from previous studies (*38*, *39*, *43*, *47*), a *P*-*T*-*X* diagram is derived to illustrate the phase relations of the Na_2_CO_3_-H_2_O system over a broad *P*-*T* area (Fig. 3A). It can be seen that the Na_2_CO_3_ concentration has a positive pressure dependence over the entire temperature range, with a much deeper slope under high-temperature and low-pressure conditions, due to the fluids being more compressible under such conditions (*48*). At *T* > 500°C, the Na_2_CO_3_ solubility curve intersects the melt-fluid immiscibility zone and splits into two parts. To the high-pressure side, it extends along the Na_2_CO_3_ hydrous melting curve from the upper critical end point around 500°C and 1.5 kbar to the Na_2_CO_3_ melting point at 851°C (*43*), implying that a Na_2_CO_3_ saturation fluid is equivalent to a hydrous Na_2_CO_3_ melt under high *P-T* conditions. To the low-pressure side, the Na_2_CO_3_ solubility curve joins the aqueous brine equilibrated with a Na_2_CO_3_ melt and is bounded by the isochore extended from the H_2_O critical point, due to the insoluble nature of Na_2_CO_3_ in vapor-like supercritical H_2_O fluids (*36*, *37*). The melt-fluid immiscibility zone in the *P*-*T*-*X* diagram displays as a half-dome shape beneath the solubility curve (Fig. 3A), with the Na_2_CO_3_ supercritical curve as its upper *P*-*T* boundary. Because of the lack of a precise and reliable pressure calibration method, the *P-T* path of the supercritical curve is not determined precisely in the present study. However, it is estimated to have a positive d*P*/d*T* slope and stretches from the upper critical end point around 500°C and 1.5 kbar to 4.6 to 5.4 kbar at 700°C, as melt-fluid immiscibility occurred in load #5 but not in load #7.

To better illustrate the phase changes in the Na_2_CO_3_-H_2_O system under different *P*-*T* conditions, four isobaric sections of the *P*-*T*-*X* diagram at 0.75, 1.5, 3.0, and 4.5 kbar are shown in Fig. 3 (B to E, respectively). The 0.75-kbar section (Fig. 3B) is characterized by a retrograde Na_2_CO_3_ solubility curve, and the occurrence of a Na_2_CO_3_ melt either with natrite solids (S + M) or with a vapor-like supercritical fluid (M + V, fluid density is lower than the H_2_O critical point), which implies the release of H_2_O as steam from carbonatitic subvolcanoes. The 1.5-kbar section (Fig. 3C) is unique, in which the Na_2_CO_3_ solubility remains constant and the Na_2_CO_3_ solubility and melting curves intersect around the upper critical end point (*40*, *41*). The phase relation at the upper critical end point would be natrite solid with a supercritical liquid (currently not observed), which subsequently separates into a Na_2_CO_3_ brine-melt and an aqueous fluid with a further increase in temperature (S + BM + L, fluid density is higher than the H_2_O critical point). Meanwhile, the melt-fluid immiscibility curve deviates from the solubility curve and lies beneath it, leading to occurrence of the Na_2_CO_3_ brine-melt either as a single phase (BM, low H_2_O), or equilibrated with a natrite solid (S + BM, very low H_2_O) or an aqueous fluid (BM + L, low to high H_2_O), or absence of the Na_2_CO_3_ brine-melt but an aqueous fluid with limited Na_2_CO_3_ concentration (L, very high H_2_O) instead. Under higher pressures (e.g., 3.0 kbar; Fig. 3D), the Na_2_CO_3_ solubility curve becomes intensely prograde, which results in the saturation liquid at *T* < 500°C being highly concentrated and, hence, the occurrence of S + BM over a broad temperature range (Fig. 3, D and E). The BM + L immiscibility area shrinks with increasing pressure and finally disappears at *P-T* conditions exceeding the supercritical curve (deep crustal pressure; Fig. 3E). In this case, the system occurs either as coexistence of natrite with a Na_2_CO_3_ brine-melt (S + BM) or as a homogeneous liquid, which can be called a brine-melt (BM, low H_2_O) or an aqueous fluid (L, high H_2_O), with no boundary between these two phases.

## DISCUSSION

### Transitions from carbonatitic brine-melts to hydrothermal brines

The two distinct melt-fluid transition processes in the Na_2_CO_3_-H_2_O system under deep and shallow crustal conditions reveal that hydrothermal brines can be generated from continuous fractionation of carbonatitic magmas or through fluid exsolution, depending on whether the *P*-*T* condition is greater or not than the supercritical curve of the Na_2_CO_3_-H_2_O system. Estimated emplacement depths of carbonatites span a wide range from extrusive volcanoes to deep-seated intrusions (*10*), indicating that both continuous melt-fluid transition and fluid exsolution could occur during the transition from carbonatitic magmas to hydrothermal brines. To be specific, in deep-seated carbonatitic intrusions, such as the Mianning-Dechang REE belt (Maoniuping, Dalucao, Muluozhai, and Lizhuang) (*18*, *42*, *45*), the Okorusu fluorite deposit (*29*), the Evate carbonatite (*44*), and many other carbonatite complexes worldwide (*8*, *10*, *26*, *49*), the trapping conditions of melt-fluid inclusions (600° to 850°C, 3.0 to 15.7 kbar) were clearly above the critical curve of the Na_2_CO_3_-H_2_O system, suggesting that the melt-fluid evolution processes in these carbonatites were continuous without exsolution of synmagmatic brines. The abundance of daughter minerals (40 to 80 volume percent) and very high salinities [up to 80 wt % NaCl-equivalent (NaCleq)] in the melt-fluid inclusions (*10*, *18*, *26*, *29*, *45*, *49*) yield the development of carbonatitic pegmatites and mineralization of REEs from highly evolved brine-melts as well. By contrast, the synmagmatic brines released from shallow intrusive carbonatites, such as the Amba Dongar carbonatite (*50*), the Siilinjärvi carbonatite complex (*51*), and the Songwe Hill carbonatite (*52*), were characterized by simple NaCl-H_2_O-CO_2_ bulk compositions with <20 wt % salinities, consistent with the aqueous fluids exsolved from carbonatitic melts under shallow crustal conditions. The estimated *P*-*T* conditions for fluid exsolution (~620°C, 1.0 to 3.0 kbar) located well in the melt-fluid immiscibility zone (Fig. 3A), further supporting that these brines were released from the magmas through fluid-melt immiscibility.

It should be noted here that owing to the abundance of other salt species and volatiles in carbonatitic magmas, the fluid composition and *P*-*T* condition for melt-fluid immiscibility could vary greatly from the Na_2_CO_3_-H_2_O binary system. For instance, both CaCO_3_ and K_2_CO_3_ are major components in highly evolved Na-rich carbonatitic magmas (*2*, *6*–*8*, *12*); hence, the formation of Na-K-Ca carbonate minerals (nyerereite, gregoryite, fairchildite, etc.) at *T* < 600°C (*12*) could suppress the mobilization of alkalis in aqueous brines or brine-melts under shallow and deep crustal conditions, respectively. Likewise, the effect of silica from the surrounding rocks is also prominent, not only lowering the solidus of the Na_2_CO_3_ melt by over 100°C (*40*) but also inhibiting the mobilization of alkalis and REEs through precipitation of alkali-silicate minerals from the brine-melts (*11*). Furthermore, the abundance of CO_2_ reduces the miscibility between carbonatitic melts and H_2_O-CO_2_ fluids, which enlarges the immiscibility zone to higher *P*-*T* conditions (*53*), while that of F^−^ and SO_4_^2−^ potentially enhances the decrease in alkali concentrations during cooling and decompression of the aqueous brines or brine-melts (*35*, *41*). To summarize, the *P-T-X* properties of the Na_2_CO_3_-H_2_O system establish a basic framework for interpreting the magmatic hydrothermal processes in carbonatites, but the effects from other dissolved components also need to be considered with caution.

### REE mineralization at the absence and presence of melt-fluid immiscibility in carbonatites

Among the more than 600 carbonatites on our planet, only less than 30 display potentially economic mineralization of REEs, and investigations of the REE mineralization processes were carried out thoroughly just in a few particular deposits (*3*). Because of the incompatible nature of REEs in fluorite, calcite, and dolomite (*15*–*17*, *32*), the prolonged fractional crystallization of fluorite and Ca-, Mg-, or Fe-carbonate minerals under deep crustal conditions results in sufficient enrichment of REEs in the brine-melts to form alkali REE carbonates, such as burbankite or carbocernaite group minerals, which subsequently alter into ancylite, bastnäsite with synchysite, or parisite by postmagmatic fluids (*14*). This REE mineralization type has been extensively recognized in the Bear Lodge (*31*), Palabora (*24*), Vuoriyarvi (*54*), and other carbonatites (*30*, *32*), where the abundance of multiphase melt-fluid inclusions implies the occurrence of carbonatitic brine-melts under deep crustal conditions (*10*, *55*, *56*). While the carbonatitic magmas or brine-melts are contaminated by silicate wall rocks, as with the cases in the Tuva REE-rich ferrocarbonatite and the Maoniuping and Mountain Pass REE deposits, the crystallization of alkali-silicate minerals (aegirine, arfvedsonite, phlogopite, alkali feldspar, etc.) suppresses the precipitation of alkali REE carbonates (*11*, *14*), leading to the formation of magmatic bastnäsite and parisite instead (*3*, *8*, *18*, *33*). In these scenarios, the continuous melt-to-fluid inclusion spectra record the evolution from high *P-T* (650° to 800°C, >3.5 kbar) brine-melts enriched in alkali carbonates, sulfates, and REEs to hydrothermal brines with moderate temperatures (300° to 400°C) and salinities (10 to 30 wt % NaCleq) after the precipitation of REE minerals (*8*, *18*, *42*), further supporting the mineralization of REEs from carbonatitic brine-melts under deep crustal conditions. Note that the continuous melt-fluid evolution does not preclude the mineralization of REEs as phosphates from the brine-melts (*8*, *31*, *33*), while the primary REE minerals (particularly the burbankite or carbocernaite group minerals) are commonly altered by postmagmatic hydrothermal activities along with brecciation or hydraulic fracturing of the country rocks (*1*, *9*, *10*), which further complicate interpretation of the geological information recorded in mineral paragenesis and/or fluid inclusions.

While emplacement of carbonatitic magmas occurs at shallow crustal levels, exsolution of synmagmatic brines occurs under high-temperature but low-pressure conditions, with REEs being largely retained in the melts (*21*, *22*) and deposit as magmatic minerals (e.g., apatite and monazite). In these scenarios, the enrichment of REEs is terminated by solidification of the carbonatitic melts (Fig. 3C), leading to REEs dispersing in the carbonatitic rocks instead of forming high-grade economic REE ore bodies. Such REE mineralization type has been reported at Amba Dongar (*23*), Siilinjärvi (*57*), and Songwe Hill carbonatites (*52*), all of which are characterized by low fluid exsolution pressures (1.0 to 3.0 kbar), and simple NaCl-H_2_O-CO_2_ fluid compositions with salinities no greater than 20 wt % NaCleq (*50*–*52*). Although alteration of the magmatic REE minerals during postmagmatic hydrothermal activities leads to precipitations of bastnäsite, parisite, and synchysite in and around the carbonatites (*23*, *31*, *52*), such secondary REE mineralization takes place in limited scales and commonly with non- or subeconomic interests under current exploring or mining techniques.

## MATERIALS AND METHODS

All samples in our experiments were subjected to high *P-T* conditions using a Bassett-type (*58*, *59*) hydrothermal diamond anvil cell (HDAC, type V). To avoid fluid leakage and chemical reactions between the rhenium gasket and the carbonate solution under high-temperature conditions, the sample chamber hole in the gasket was preindented and lined by a 50-μm-thick gold layer. Ten Na_2_CO_3_-H_2_O loads (#1 to 3 and #6 to 12) were prepared by loading analytical grade anhydrous Na_2_CO_3_ pellets (Na_2_CO_3_ ≥ 99.9 wt %) with deionized water into HDAC under room *P-T *conditions, together with air bubbles to control the *P-T* paths of the fluids (Figs. 1A, a, and 2A, a). Four additional loads were prepared by loading Na_2_CO_3_ with K_2_CO_3_ solution (1 mol/kg; #4 and 13) or with H_2_O and Iceland spar fragments (#5 and 14), to investigate the effects from K_2_CO_3_ or CaCO_3_ on the phase transitions and the fluid or melt compositions under different *P-T* conditions. During the experiment, each sample was heated at ~5°C/min, with Raman spectrum of the solution being measured after a 5-min suspension at each temperature point with intervals of 50°C. Because of the minerals used for pressure calibration in HDAC experiments (quartz, zircon, anhydrite, etc.) (*60*–*62*) becoming extremely soluble in Na_2_CO_3_ solutions at *T* > 400°C, fluid pressure in the Na_2_CO_3_-H_2_O (#1 to 3 and 6 to 12) and Na_2_CO_3_-K_2_CO_3_-H_2_O (#4 and 13) loads was estimated from the liquid-vapor homogenization temperature using isochoric *P-T* curves defined for the NaCl-H_2_O system (*63*), on the basis that Na_2_CO_3_ fluids have similar densities to NaCl ones (e.g., <0.02 g/cm^3^ at 400°C and 5.0 kbar) with equivalent mass concentrations (*48*). In the Na_2_CO_3_ + CaCO_3_ + H_2_O loads (#5 and 14), error between the fluid pressures as calibrated using the microthermometric method and from the frequency shift in the ν_1, 1086_ Raman band of calcite (*64*) varied less than ±0.4 kbar. The last two loads (#15 and 16) were prepared to investigate the effect of pressure on Na_2_CO_3_ solubility under 120 and 200°C, where fluid pressure was calibrated from frequency shift in the 464 cm^−1^ Raman band of quartz (*61*), with the solubility of quartz being estimated to be <0.1 mol/kg (*65*).

Unpolarized Raman spectra of Na_2_CO_3_ solutions were excited by a 532-nm Neodymium-doped Yttrium Aluminium Garnet (Nd-YAG) laser (100 mW) and collected using a Renishaw inVia Raman spectrometer installed at the Institute of Mineral Resources, Chinese Academy of Geological Sciences. The spectrometer is equipped with a Leica ×10 long-working distance objective (numerical aperture = 0.25), a 2400 gr/mm grating and a 1024 × 256 pixel charge-coupled device detector, providing a spectral pixel resolution of 1.2 cm^−1^. Each spectrum was acquired with five accumulations of 10 s each in the 100 to 4200 cm^−1^ spectral range, with the focus position of the objective being fixed ~20 μm below the interface between fluid and upper diamond anvil (*59*). The symmetric stretching vibrations of CO_3_^2−^ (ν_1_) and HCO_3_^−^ (ν_5_) were fitted using Gaussian + Lorentzian profiles, after baseline corrections using quadratic curves to remove the interference from the ν_1_ band of diamond; the O-H stretching bands of water were fitted using three Gaussian subbands, after baseline corrections using a linear function (fig. S4) (*46*).

Raman quantification for CO_3_^2−^ concentration in high *P-T* aqueous fluids was carried out using the integrated peak intensity of the O-H band of water (sum of three subbands) as an internal standard (*66*). The variation in CO_3_^2−^-to-H_2_O Raman peak intensity ratio (*R*_intensity_) with temperature and CO_3_^2−^ concentration was calibrated in advance, by using the same instrument and 1.03 to 18.80 mol/kg (12.45 to 72.18 wt %) K_2_CO_3_ solutions in the 23° to 700°C temperature range (calibration procedures are included in the Supplementary Materials). The CO_3_^2−^-to-HCO_3_^−^ Raman scattering factor ratio has been shown to vary less than 10% from 1.46 at *P*-*T* conditions up to 600°C and 1 GPa (*65*, *67*); therefore, in the spectra where both CO_3_^2−^ and HCO_3_^−^ Raman bands were distinguishable, the HCO_3_^−^ peak amplitude was corrected by a factor of 1.46, and *R*_intensity_ was calculated from the ratio of combined CO_3_^2−^ and HCO_3_^−^ Raman peak amplitudes to that of the O-H band of water. According to the fitting results from our calibration experiment (tables S3 to S5 and fig. S6), error in Raman quantification results for CO_3_^2−^ concentration in a high *P-T* alkali carbonate (K_2_CO_3_, Na_2_CO_3_, or Cs_2_CO_3_) solution was generally below ±0.3 mol/kg.

## Supplementary Materials

### This PDF file includes:

Supplementary Text Figs. S1 to S6 Tables S1 to S5 References
