# Supplementary material for: Transition from carbonatitic magmas to hydrothermal brines: Continuous dilution or fluid exsolution?
Source: Sci Adv. 2023 Jul 19;9(29):eadh0458. doi: 10.1126/sciadv.adh0458 (PMC10355818; doi:10.1126/sciadv.adh0458)
Supplement: Supplementary file 1 — Supplementary Text Figs. S1 to S6 Tables S1 to S5 References [file sciadv.adh0458_sm.pdf]

Science Advances
AAAS

Supplementary Materials for

Transition from carbonatitic magmas to hydrothermal brines: Continuous dilution or fluid exsolution?

Xueyin Yuan *et al.*

Corresponding author: Xueyin Yuan, xueyinyuan@live.com; Richen Zhong, zhongrichen@126.com

Sci. Adv. **9**, eadh0458 (2023)
DOI: 10.1126/sciadv.adh0458

This PDF file includes:

Supplementary Text
Figs. S1 to S6
Tables S1 to S5
References

Supplementary Text

RESULTS

Phase transitions in the Na_2CO_3-$CaCO_3$-H_2O system under shallow and deep crustal conditions are shown in Figures S1 and S2, respectively. The addition of calcite resulted in the formation of nyerereite [$Na_2Ca(CO_3]_2$] between 300 and 600 °C, but didn't prohibit the increase in Na_2CO_3 solubility with increasing *P-T* condition, nor the exsolution of synmagmatic brines from shallow carbonatitic magmas bring replaced by continuous melt-fluid transition in the deep crust.

Raman quantification results for integrated CO_3^{2-} concentration in Na_2CO_3-saturated aqueous fluids or brine-melts under high *P-T* conditions are listed in Table S1.

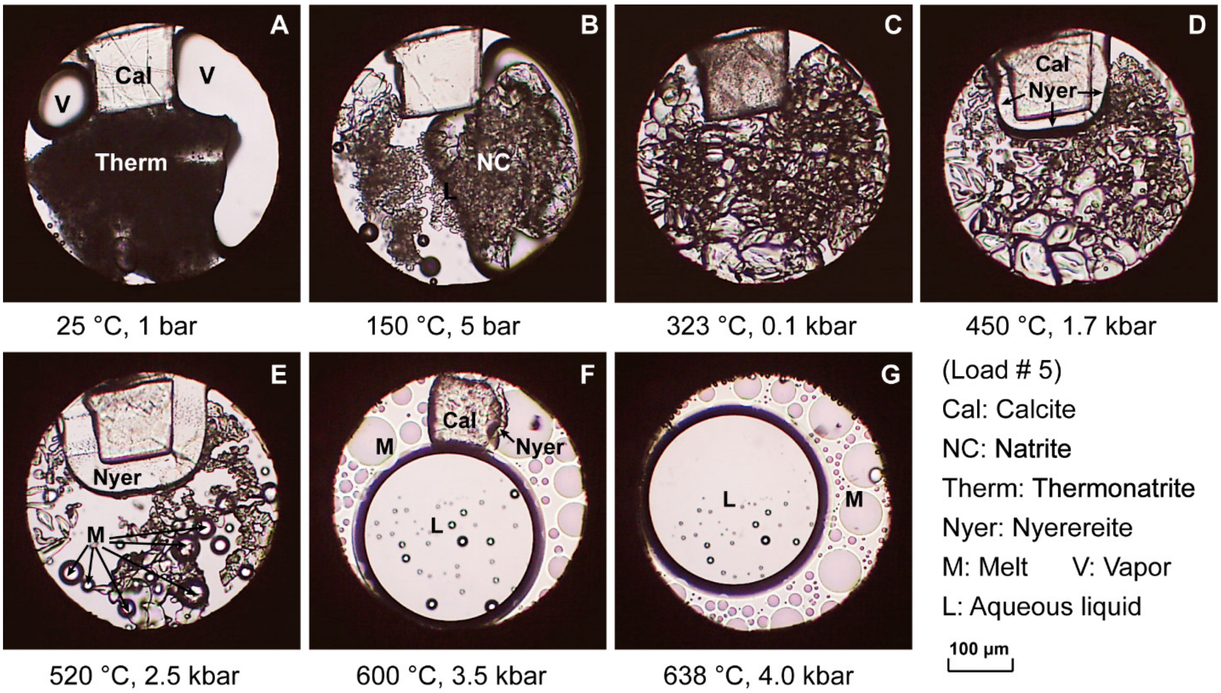

Figure S1 Photomicrographs showing the phase changes in the Na_2CO_3-$CaCO_3$-H_2O system under shallow crustal conditions. Calcite between 300 and 600 °C was covered by a nyerereite shell, Na_2CO_3 melt was observed at *P-T* conditions above 500 °C and 2.2 kbar.

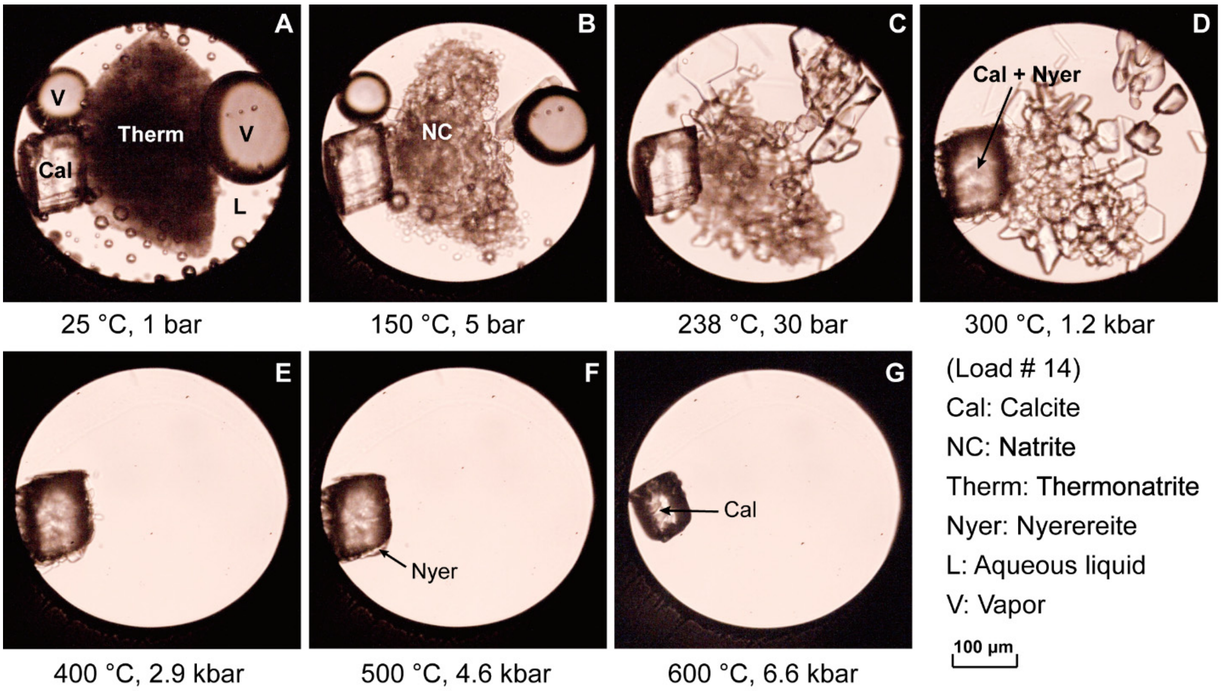

Figure S2 Photomicrographs showing the phase changes in the Na_2CO_3-$CaCO_3$-H_2O system under deep crustal conditions. Calcite in the 300 – 500 °C temperature range was covered by nyerereite microcrystals.

Table S1 Raman quantification results for integrated CO_3^{2-} concentration in Na_2CO_3-saturated aqueous fluids or brine-melts under high *P*-*T* conditions.

Temperature	Pressure [a]	Raman peak intensity				CO_3^{2-} concentration		Density
		CO_3^{2-}	HCO^-	H_2O	$R_{intensity}$ [b]	mol/kg	Wt.%	(g/cm^3) [c]
**Load #1** (Na_2CO_3+H_2O, Th_{L-V} 376 °C, Tm_{NC} 530 °C, liquid-melt homogenization not achieved) [d]								
50 °C	1 bar	8738	N.D. [e]	8695	1.005	4.42	31.90	1.343
75 °C	1 bar	8030	N.D.	8457	0.949	4.21	30.87	1.318
100 °C	1 bar	6603	N.D.	7139	0.925	4.16	30.58	1.300
150 °C	5 bar	5566	771	8704	0.769	3.55	27.33	1.223
200 °C	16 bar	4240	708	9183	0.574	2.76	22.61	1.118
250 °C	40 bar	2484	569	8491	0.390	2.01	17.58	0.996
300 °C	84 bar	895	469	8484	0.186	1.16	10.97	0.840
350 °C	0.2 kbar	239	192	8043	0.065	0.74	7.25	0.668
400 °C	0.4 kbar	N.D.	N.D.	11855	N.D.	N.D.	N.D.	0.322
450 °C	0.8 kbar	N.D.	N.D.	13012	N.D.	N.D.	N.D.	0.322
500 °C	1.3 kbar	288	444	16345	0.057	1.57	14.30	0.523
550 °C	1.7 kbar	245	675	14556	0.085	2.12	18.38	0.585
600 °C	2.2 kbar	326	835	24737	0.063	2.43	20.49	0.618
650 °C	2.7 kbar	343	1016	16532	0.111	3.22	25.44	0.700
700 °C	3.2 kbar	255	1216	18032	0.113	3.77	28.58	0.755
**Load #2** (Na_2CO_3+H_2O, Th_{L-V} 355 °C, Tm_{NC} 500 °C, liquid-melt homogenized at 709 °C)								
200 °C	16 bar	3489	837	7531	0.625	3.01	24.18	1.138
250 °C	40 bar	3283	966	10592	0.443	2.28	19.45	1.020
300 °C	84 bar	2244	970	10167	0.270	1.59	14.44	0.880
350 °C	0.2 kbar	890	746	10487	0.113	0.99	9.53	0.694
400 °C	0.6 kbar	1277	902	11584	0.224	1.85	16.39	0.781
450 °C	1.1 kbar	2035	802	10743	0.298	2.59	21.55	0.853
500 °C	1.6 kbar	702	502	10119	0.142	2.06	17.94	0.801
550 °C	2.2 kbar	708	994	14692	0.147	2.50	20.92	0.843
600 °C	2.7 kbar	747	1567	25558	0.119	2.78	22.73	0.868
650 °C	3.3 kbar	906	1690	25982	0.130	3.34	26.15	0.919

709 °C	4.0 kbar	11490	690	7977	1.567	13.52	58.90	1.511
**Load #3** (Na_2CO_3+H_2O, Th_{L-V} 345 °C, Tm_{NC} 500 °C, liquid-melt homogenization not achieved)								
100 °C	1 bar	11680	N.D.	12429	0.940	4.23	30.94	1.305
150 °C	5 bar	8085	1113	12153	0.799	3.69	28.14	1.233
200 °C	15 bar	4862	1084	11817	0.545	2.61	21.70	1.106
250 °C	40 bar	2443	571	8436	0.389	2.00	17.51	0.996
300 °C	86 bar	1014	403	10115	0.159	1.02	9.76	0.826
350 °C	0.2 kbar	515	461	9995	0.119	1.02	9.79	0.698
400 °C	0.6 kbar	942	555	9855	0.178	1.60	14.49	0.756
450 °C	1.1 kbar	1454	675	9013	0.271	2.43	20.52	0.838
500 °C	1.6 kbar	515	514	10099	0.125	1.97	17.25	0.792
550 °C	2.2 kbar	534	574	9680	0.142	2.46	20.71	0.840
600 °C	2.7 kbar	666	693	11450	0.147	2.94	23.79	0.884
650 °C	3.3 kbar	667	520	9979	0.143	3.42	26.62	0.926
700 °C	4.0 kbar	361	360	9817	0.090	3.63	27.79	0.944
**Load #4** (Na_2CO_3+K_2CO_3+H_2O, Th_{L-V} 352 °C, Tm_{NC} 450 °C, liquid-melt homogenization not achieved)								
100 °C	1 bar	14531	N.D.	15681	0.927	4.16	30.62	1.301
150 °C	5 bar	11519	364	14394	0.837	3.88	29.14	1.246
200 °C	16 bar	9477	414	14189	0.711	3.43	26.66	1.171
250 °C	40 bar	7183	422	12743	0.612	3.13	24.94	1.091
300 °C	70 bar	5610	406	13354	0.465	2.60	21.61	0.973
350 °C	0.2 kbar	3998	460	14693	0.318	2.08	18.06	0.799
400 °C	0.6 kbar	3967	447	13485	0.343	2.50	20.96	0.840
450 °C	1.1 kbar	1324	551	11401	0.187	1.96	17.20	0.786
500 °C	1.6 kbar	1146	658	13692	0.154	2.13	18.43	0.803
550 °C	2.1 kbar	879	598	12454	0.141	2.46	20.67	0.834
600 °C	2.6 kbar	698	430	12086	0.110	2.72	22.38	0.858
**Load #5** (Na_2CO_3+H_2O+Calcite, Th_{L-V} 323 °C, Tm_{NC} 530 °C, liquid-melt homogenization not achieved)								
100 °C	1 bar	12231	N.D.	13580	0.901	4.04	29.99	1.292
150 °C	5 bar	5832	654	9070	0.748	3.45	26.78	1.215
200 °C	16 bar	4716	929	10219	0.594	2.85	23.23	1.126
250 °C	40 bar	3073	565	10477	0.372	1.92	16.91	0.988

300 °C	70 bar	1397	482	11658	0.180	1.13	10.71	0.837
350 °C	0.3 kbar	805	378	12026	0.113	0.99	9.51	0.781
400 °C	0.9 kbar	767	413	10453	0.131	1.34	12.47	0.815
450 °C	1.7 kbar	997	302	10936	0.132	1.65	14.88	0.844
500 °C	2.2 kbar	874	454	11425	0.134	2.02	17.63	0.878
550 °C	2.8 kbar	816	422	13333	0.107	2.26	19.33	0.900
600 °C	3.4 kbar	435	421	11835	0.089	2.59	21.55	0.930
**Load #6** (Na_2CO_3+H_2O, Th_{L-V} 253 °C, Th_{NC} 462 °C)								
39 °C	1 bar	8695	N.D.	8251	1.054	4.62	32.89	1.360
63 °C	1 bar	7385	N.D.	7606	0.971	4.29	31.25	1.329
87 °C	1 bar	7291	N.D.	7830	0.931	4.15	30.57	1.307
100 °C	1 bar	5952	N.D.	6368	0.935	4.20	30.81	1.304
125 °C	2 bar	6439	N.D.	7477	0.861	3.92	29.35	1.271
150 °C	5 bar	5166	394	7511	0.764	3.53	27.22	1.221
175 °C	9 bar	4368	465	7325	0.689	3.24	25.55	1.176
200 °C	16 bar	3719	496	7576	0.586	2.82	22.99	1.123
225 °C	25 bar	2902	395	7112	0.489	2.42	20.41	1.068
250 °C	40 bar	1731	385	6274	0.365	1.89	16.67	0.985
275 °C	0.3 kbar	1932	416	6598	0.385	2.08	18.07	0.999
300 °C	0.7 kbar	2491	498	6263	0.514	2.86	23.26	1.065
350 °C	1.6 kbar	4805	447	6208	0.879	4.64	32.98	1.201
400 °C	2.5 kbar	6979	338	6298	1.186	6.59	41.13	1.326
450 °C	3.5 kbar	8757	272	6022	1.520	9.77	50.89	1.487
463 °C	3.7 kbar	9203	201	5961	1.593	10.37	52.36	1.512
500 °C	4.4 kbar	8735	199	5784	1.560	10.61	52.94	1.521
**Load #7** (Na_2CO_3+H_2O, Th_{L-V} 298 °C, Th_{NC} 495 °C)								
50 °C	1 bar	6749	N.D.	6754	0.999	4.39	31.78	1.341
75 °C	1 bar	6542	N.D.	6928	0.944	4.19	30.75	1.316
100 °C	1 bar	6262	N.D.	6681	0.937	4.21	30.88	1.304
150 °C	5 bar	4998	498	7481	0.765	3.53	27.24	1.221
200 °C	16 bar	4740	579	9450	0.591	2.84	23.13	1.125
250 °C	40 bar	2634	625	8775	0.404	2.08	18.08	1.003

300 °C	70 bar	1555	584	9353	0.257	1.53	13.94	0.874
350 °C	0.7 kbar	1864	508	7856	0.332	2.15	18.58	0.932
400 °C	1.4 kbar	3276	689	8224	0.521	3.49	27.00	1.047
450 °C	2.1 kbar	4737	593	6769	0.828	5.64	37.42	1.206
495 °C	2.8 kbar	7208	560	6323	1.270	8.75	48.11	1.387
**Load #8** (Na_2CO_3+H_2O, $Th_{L\text{-}V}$ 261 °C, Th_{NC} 480 °C)								
100 °C	1 bar	7698	N.D.	7801	0.987	4.45	32.04	1.177
200 °C	16 bar	4039	895	8664	0.617	2.97	23.92	1.067
250 °C	30 bar	3862	1241	13567	0.418	2.15	18.58	1.059
300 °C	0.8 kbar	4423	1123	12274	0.494	2.75	22.60	1.175
350 °C	1.6 kbar	6396	1063	11047	0.720	4.25	31.06	1.328
400 °C	2.4 kbar	9642	1055	10469	1.068	6.59	41.14	1.447
450 °C	3.2 kbar	10976	766	8830	1.370	8.86	48.43	1.532
480 °C	3.8 kbar	11000	493	7182	1.632	10.81	53.41	1.533
500 °C	4.1 kbar	10816	545	7252	1.601	10.87	53.54	1.320
**Load #9** (Na_2CO_3+H_2O, $Th_{L\text{-}V}$ 212 °C, Th_{NC} 555 °C)								
200 °C	16 bar	3971	1652	9888	0.646	3.11	24.78	1.137
250 °C	0.8 kbar	4273	1727	9223	0.737	3.77	28.57	1.187
300 °C	1.8 kbar	5465	1524	9337	0.824	4.50	32.28	1.237
350 °C	2.7 kbar	6802	1416	7649	1.160	6.69	41.50	1.373
400 °C	3.7 kbar	8319	1217	6975	1.447	8.80	48.27	1.480
450 °C	4.6 kbar	10946	1159	6984	1.807	11.53	55.00	1.592
500 °C	5.5 kbar	13490	1172	6495	2.341	15.61	62.33	1.719
550 °C	6.5 kbar	16280	901	6290	2.798	19.49	67.38	1.810
555 °C	6.6 kbar	19530	819	7178	2.887	20.19	68.16	1.825
**Load #10** (Na_2CO_3+H_2O, $Th_{L\text{-}V}$ 200 °C, Th_{NC} 512 °C)								
200 °C	16 bar	4422	1219	8735	0.639	3.08	24.58	1.143
250 °C	0.9 kbar	4416	1001	7293	0.725	3.71	28.25	1.191
300 °C	1.9 kbar	6263	1030	9379	0.828	4.52	32.40	1.247
350 °C	2.8 kbar	7614	952	7726	1.165	6.72	41.62	1.383
400 °C	3.7 kbar	9795	717	7447	1.456	8.85	48.41	1.489
450 °C	4.6 kbar	12760	715	7404	1.864	11.89	55.76	1.611

500 °C	5.6 kbar	13850	685	6256	2.374	15.83	62.65	1.730
512 °C	5.8 kbar	13480	653	5838	2.472	16.66	63.85	1.751
**Load #11** (Na_2CO_3+H_2O, $Th_{L\text{-}V}$ 164 °C, Th_{NC} 450 °C)								
175 °C	0.3 kbar	4764	684	7976	0.723	3.40	26.50	1.204
200 °C	0.8 kbar	4053	556	6417	0.758	3.67	27.98	1.222
250 °C	1.8 kbar	4785	525	6216	0.893	4.58	32.69	1.285
300 °C	2.8 kbar	5008	437	5246	1.076	5.86	38.30	1.364
350 °C	3.8 kbar	5942	556	5049	1.338	7.70	44.94	1.462
400 °C	4.9 kbar	8694	571	5683	1.677	10.16	51.86	1.571
450 °C	5.9 kbar	8605	536	4507	2.083	13.25	58.42	1.678
**Load #12** (Na_2CO_3+H_2O, $Th_{L\text{-}V}$ 124 °C, Th_{NC} 381 °C)								
100 °C	1 bar	9429	N.D.	9401	1.003	4.52	32.41	1.325
150 °C	0.5 kbar	8671	952	9729	0.910	4.23	30.97	1.291
200 °C	1.6 kbar	8910	1194	9762	0.960	4.68	33.15	1.320
250 °C	2.6 kbar	9254	1071	9656	1.064	5.48	36.72	1.368
300 °C	3.6 kbar	8870	1193	8479	1.252	6.81	41.94	1.442
350 °C	4.7 kbar	7989	916	5825	1.569	9.02	48.89	1.546
381 °C	5.2 kbar	11350	1180	7786	1.679	9.98	51.40	1.584
**Load #13** (Na_2CO_3+K_2CO_3+H_2O, $Th_{L\text{-}V}$ 147 °C, Th_{NC} 320 °C)								
100 °C	1 bar	11465	N.D.	12102	0.947	4.26	31.12	1.307
150 °C	20 bar	9729	452	12245	0.848	3.93	29.42	1.258
200 °C	1.2 kbar	9518	331	11345	0.882	4.28	31.22	1.280
250 °C	2.2 kbar	10383	366	11412	0.957	4.91	34.24	1.321
300 °C	3.2 kbar	10882	327	10181	1.116	6.07	39.16	1.390
320 °C	3.7 kbar	11635	331	10252	1.182	6.58	41.09	1.418
350 °C	4.3 kbar	11635	331	10252	1.182	6.58	41.09	1.417
400 °C	5.3 kbar	11239	321	10303	1.136	6.56	41.02	1.414
**Load #14** (Na_2CO_3+H_2O+Calcite, $Th_{L\text{-}V}$ 238 °C, Th_{NC} 384 °C)								
100 °C	1 bar	9921	N.D.	11119	0.892	4.00	29.79	1.290
150 °C	5 bar	6622	676	10158	0.749	3.45	26.80	1.216
200 °C	16 bar	5038	705	10729	0.566	2.71	22.34	1.114
250 °C	0.5 kbar	4132	933	10780	0.510	2.61	21.69	1.061

300 °C	1.2 kbar	4594	626	10404	0.529	2.94	23.76	1.087
350 °C	2.0 kbar	5750	630	9614	0.694	4.11	30.34	1.177
400 °C	2.9 kbar	6819	615	9805	0.787	4.99	34.58	1.237
450 °C	3.8 kbar	5724	624	9293	0.714	4.98	34.54	1.235
500 °C	4.6 kbar	4884	546	8598	0.661	5.11	35.14	1.243
550 °C	5.6 kbar	4764	566	9099	0.614	5.32	36.04	1.256
**Load #15** (Na_2CO_3+H_2O+Quartz, temperature fixed at 120 °C)								
120 °C	1.6 kbar	9055	N.D.	7985	1.134	5.15	35.30	1.402
120 °C	2.6 kbar	9975	N.D.	8950	1.115	5.05	34.88	1.420
120 °C	4.2 kbar	9042	N.D.	7482	1.209	5.50	36.84	1.481
120 °C	5.4 kbar	9171	N.D.	7442	1.232	5.62	37.31	1.508
120 °C	7.0 kbar	10710	N.D.	8761	1.223	5.57	37.12	1.531
120 °C	8.8 kbar	9001	N.D.	7712	1.167	5.30	35.99	1.540
120 °C	11.3 kbar	8181	N.D.	6835	1.197	5.45	36.60	1.567
**Load #16** (Na_2CO_3+H_2O+Quartz, temperature fixed at 200 °C)								
200 °C	4.2 kbar	7540	511	7475	1.109	5.43	36.54	1.433
200 °C	6.4 kbar	8818	527	8133	1.179	5.79	38.04	1.494
200 °C	8.2 kbar	7982	559	7384	1.192	5.86	38.30	1.525
200 °C	11.8 kbar	7184	525	6391	1.244	6.13	39.37	1.572

Note: [a] – Pressure of the fluids was estimated from isochoric *P-T* curves defined for the NaCl-H_2O system (#1 – 4, 6 – 13) (*64*), or calibrated using the peak position shifts of the 1086 cm^{-1} peak of calcite (#5 and 14) (*65*) or the 464 cm^{-1} peak of quartz (#15 and 16) (*62*);

[b] – $R_{intensity}$ was calculated from the ratio of combined Raman peak amplitudes of CO_3^{2-} and HCO_3^- (corrected by a factor of 1.46) to that of the O-H band of water (sum of the three sub-bands);

[c] – Density was calculated by extrapolating the variation in fluid density (*68*, *69*) with changes in temperature and Na_2CO_3 concentration;

[d] – Th_{L-V}, Tm_{NC}, Th_{NC} are for liquid-vapor homogenization temperature, Na_2CO_3 melting and dissolution temperatures, respectively;

[e] – N.D. is for not detected.

MATERIALS AND METHODS

The basic rationale for Raman quantification of dissolved carbonate salts in aqueous solutions using the O-H band of water as an internal standard has been described in literature (*66*, *70*) and will not be repeated here. Given that the solubility of Na_2CO_3 under high *P*-*T* conditions is much greater than that under vapor pressure (*38*, *47*), calibration for the Raman spectra over a broad CO_3^{2-} concentration range is essential in our experiment, which is achieved by measuring the Raman spectra of K_2CO_3 solutions with CO_3^{2-} concentrations between 1.03 and 18.80 mol/kg (12.45 – 72.18 wt.%) and at temperatures from ambient to 700 °C. In order to minimize the errors in Raman quantification results, our calibration experiment was carried out according to the following procedures:

(1) Preparation for K_2CO_3 solutions under ambient *P*-*T* condition

Five aqueous solutions with initial K_2CO_3 concentrations of 1.03, 2.14, 3.39, 4.84 and 6.50 mol/kg (12.45, 22.80, 31.87, 40.05 and 47.29 wt.%), respectively, were prepared from analytical grade anhydrous K_2CO_3 reagent ($K_2CO_3 \geq 99.0\%$, dried at 150 °C) and deionized water under room temperature (21 °C). Considering that salt concentration may increase due to water evaporation while loading the K_2CO_3 solutions into HDAC, an additional calibration step was carried out under ambient *P*-*T* condition before loading the solutions into HDAC. In this step, Raman spectra of the K_2CO_3 solutions were measured by loading the solutions into silica capillary tubes (0.45, 0.15 and 100 mm in internal diameter, wall thickness and length, respectively). Based on the fitting results of the Raman spectra (Fig. S3 and Table S2), with increase in K_2CO_3 concentration from 1.03 to 6.50 mol/kg, $R_{\text{intensity}}$ and R_{area} increased linearly from 0.231 to 1.465 and from 0.011 to 0.058, respectively. Accordingly, the CO_3^{2-} concentration (c_{cal}, in mol/kg) in the K_2CO_3 solutions can be calibrated using either of the following two equations:

$c_{cal} = 4.42\ (2) \times R_{intensity}$, $R^2 = 0.9996$ (Eq. 1a)

$c_{cal} = 10.9\ (1) \times R_{area}$, $R^2 = 0.9971$ (Eq. 1b)

The K_2CO_3 solutions were then loaded into the HDAC sample chamber under ambient condition, and calibration for K_2CO_3 concentration was carried before measuring the Raman spectra under high temperature conditions. Our results show that the CO_3^{2-} concentrations increased slightly to 1.29, 2.36, 3.52, 5.14 and 6.63 mol/kg (15.11, 24.57, 32.69, 41.50 and 47.78 wt.%), respectively (Table S2), indicating that increase in salt concentration did occur while loading the solutions into HDAC.

Three oversaturated K_2CO_3 solutions were prepared by loading the 6.50 mol/kg solution together with anhydrous K_2CO_3 pellets into HDAC under ambient condition. In these solutions, the K_2CO_3 saturation concentration calibrated using $R_{intensity}$ (8.04 mol/kg) or R_{area} (8.17 mol/kg) is in nice agreement with the value (8.12 mol/kg) reported in literature (*34*). Based on the solid dissolution temperature (74, 122 and 189 °C) and the high temperature K_2CO_3 solubility curve (*34*), the K_2CO_3 concentration in these solutions was 9.86, 12.72 and 18.80 mol/kg (57.64, 63.71 and 72.18 wt.%), respectively. The influence of pressure on the Raman peak intensity of alkali carbonate solutions has been shown to be insignificant (*53*), hence was neglected in the calibration experiment.

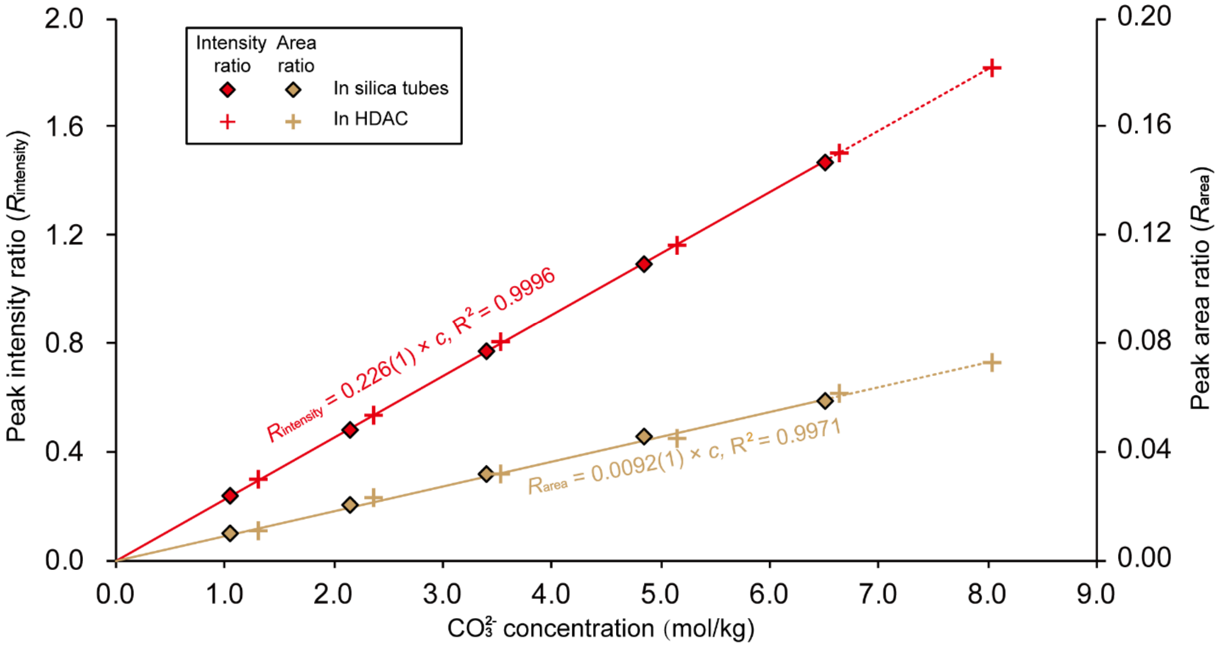

Figure S3 Variations in the CO_3^{2-}/H_2O Raman peak intensity (*$R_{intensity}$*, left vertical axis) and area (*R_{area}*, right vertical axis) ratios with K_2CO_3 concentration under ambient *P-T* condition.

Table S2 Fitting results of the CO_3^{2-} and H_2O Raman bands as measured with K_2CO_3 solutions in silica tubes and in HDAC under 24 °C and 0.1 MPa

	K_2CO_3 concentration (mol/kg)	CO_3^{2-} Raman band			H_2O Raman band [a]		$R_{intensity}$	R_{area}	c_{cal} [b] (mol/kg)
		Position	Intensity	Area	Intensity	Area			
In silica tubes	1.03	1066.47	11764	1.621E+05	50969	1.476E+07	0.231	0.011	
	2.14	1065.96	21213	3.118E+05	46689	1.462E+07	0.454	0.021	
	3.39	1065.48	34745	4.894E+05	45436	1.485E+07	0.765	0.033	
	4.84	1064.87	46355	6.574E+05	42423	1.466E+07	1.093	0.045	
	6.50	1064.26	53390	8.021E+05	36443	1.381E+07	1.465	0.058	
In HDAC	1.03	1066.60	2628	3.231E+04	9041	2.504E+06	0.291	0.013	1.29
	2.14	1066.01	4604	6.152E+04	8635	2.480E+06	0.533	0.025	2.36
	3.39	1065.87	6171	8.138E+04	7749	2.430E+06	0.796	0.034	3.52
	4.84	1065.25	10550	1.472E+05	9075	3.145E+06	1.163	0.047	5.14
	6.50	1065.16	11744	1.737E+05	7828	2.718E+06	1.507	0.064	6.63
	8.12 [c]	1063.76	12492	1.994E+05	6873	2.659E+06	1.818	0.075	8.04

Note: [a] – H_2O Raman peak intensity and area were calculated by summing the intensities and areas of the three sub-bands;

[b] – CO_3^{2-} concentrations were calculated from $R_{intensity}$ using Equation 1a;

[c] – CO_3^{2-} concentration is calculated based on the solubility of K_2CO_3 at 26 °C (*34*).

(2) Quantifying the variations in $R_{intensity}$ and R_{area} with K_2CO_3 concentration and temperature

Fittings of the CO_3^{2-} and H_2O Raman bands under ambient and high temperature conditions are shown in Figure S4. With increasing temperature, the CO_3^{2-} Raman band in K_2CO_3 solutions underwent apparent decrease in peak intensity and broadening in width. Meanwhile, the O-H stretching band of water also displayed progressive evolutions due to the breaking of the water cluster structure with increasing temperature (*71*). A weak band occurring around 980 cm^{-1} emerged above 400 °C, which indicates the presence of HCO_3^- as generated by hydrolysis between CO_3^{2-} and H_2O. In these cases, the HCO_3^- Raman peak intensity and area were converted into the equivalent values of CO_3^{2-} based on molar Raman scattering coefficients ratio of 1.46, which has been confirmed to vary less than 10% under *P*-*T* conditions up to 600 °C and 1.0 GPa (*53*, *67*), Accordingly, $R_{intensity}$ and R_{area} were calculated from the ratios of integrated Raman peak intensities and areas of CO_3^{2-} and HCO_3^-, to sum of the three sub-bands of water (Table S3).

The variations in $R_{intensity}$ and R_{area} with K_2CO_3 concentration and temperature are shown in Figure S5. From which we can see $R_{intensity}$ decreased quasi-linearly with increasing temperature, with averaged shifting rate in $R_{intensity}$ with CO_3^{2-} concentration decreasing from 0.226 at 25 °C to 0.144 at 700 °C (Fig. S5A). By contrast, R_{area} varied more irregularly between 0.005 and 0.153, and was seen to increase slowly at temperatures below 200 °C, then decreased at above 400 °C (Fig. S5B). Therefore, calibration for CO_3^{2-} concentration (c_{cal}, in mol/kg) in high temperature (T, in °C) aqueous solutions using $R_{intensity}$ yields more precise results (Fig. S6):

$$c_{cal} = 0.12(6) - 2.59(2)\times10^{-3}\times T + 4.04(4)\times R_{intensity} + 9.7(3)\times10^{-6}\times T^2 + 0.17(1)\times R^2_{intensity} + 3.39(7)\times 10^{-3}\times T\times R_{intensity},\ R^2 = 0.9994 \quad \text{(Eq. 2)}$$

Our fitting results show that errors in the Raman quantification results using $R_{intensity}$ and Eq. 2 are generally within ±0.3 mol/kg (Fig. S6), provided the CO_3^{2-} Raman peak is intense enough to be fitted precisely.

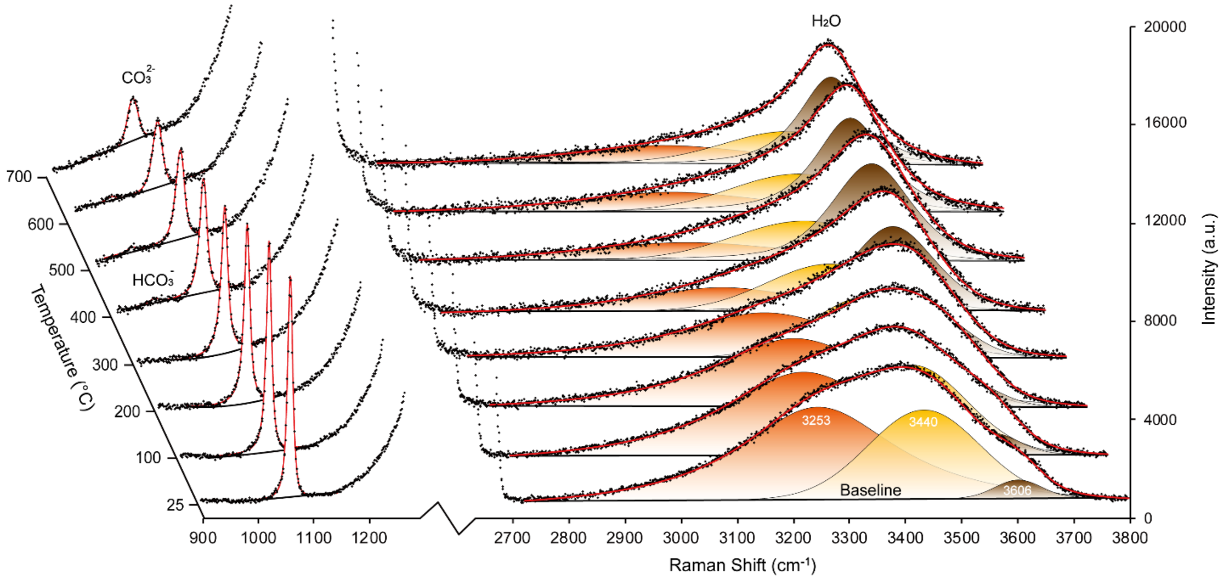

Figure S4 Fittings of the Raman spectra of K_2CO_3 solution (5.14 mol/kg, after baseline correction) measured from 25 °C to 700 °C. The CO_3^{2-} and H_2O Raman bands were fitted using 1 Gaussian + Lorentzian and 3 Gaussian profiles, respectively.

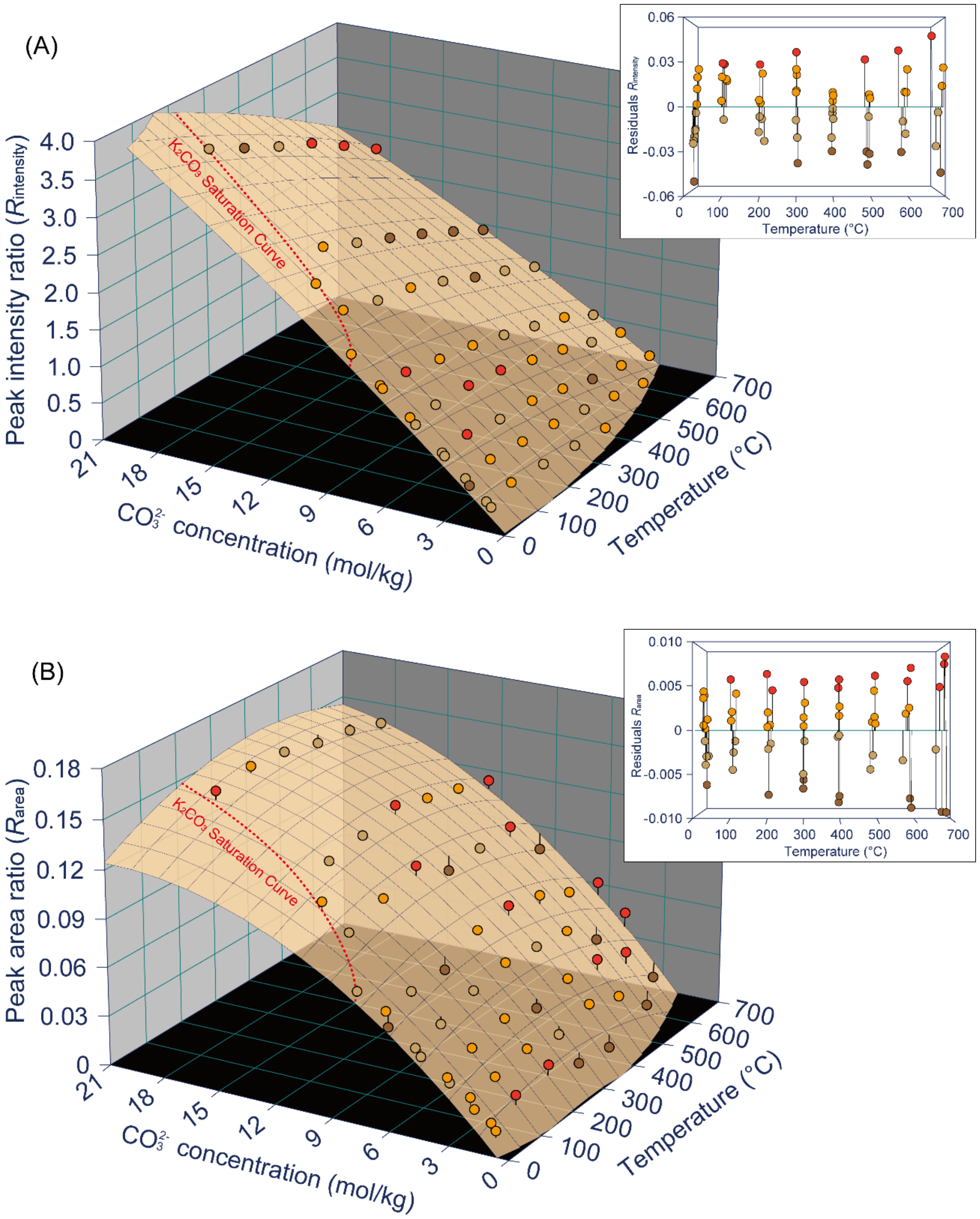

Figure S5 Variations in (A) $R_{intensity}$ and (B) R_{area} with simultaneous changes in temperature and CO_3^{2-} concentration. Colors of the data points represent the fitting residuals, as shown in the insets.

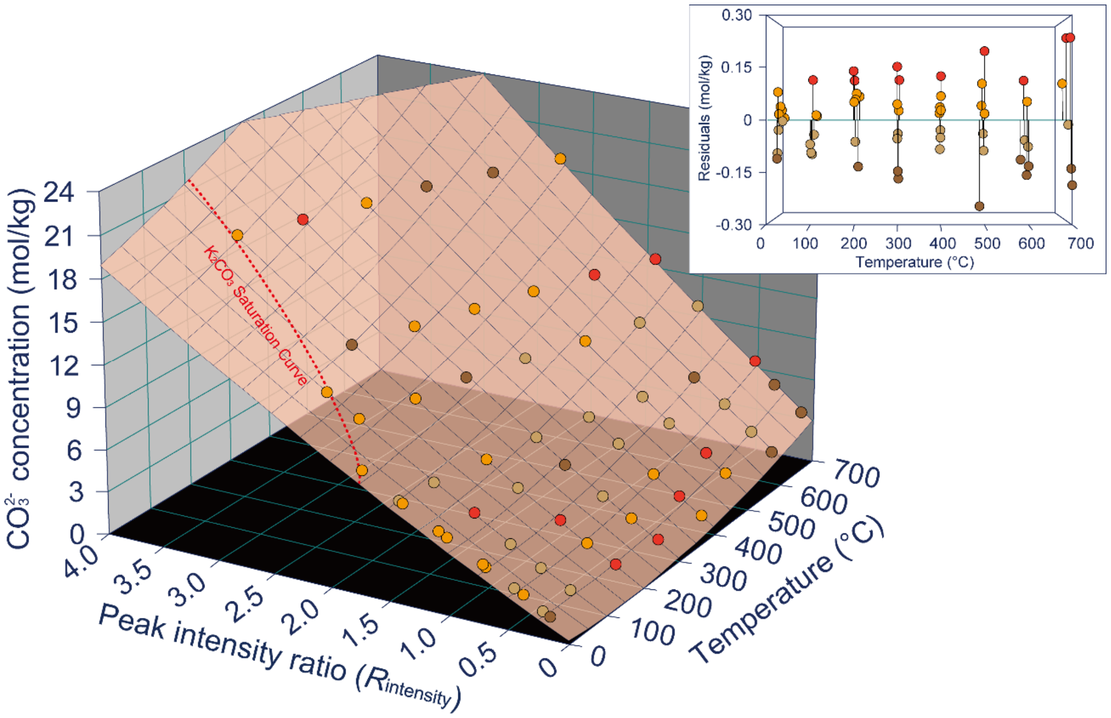

Figure S6 Fitting of the CO_3^{2-} concentration as a 2nd order polynomial function of $R_{intensity}$ and temperature. Residuals in the fitting results are shown in the inset.

Table S3 Fitting results of the CO_3^{2-} and H_2O Raman bands measured with K_2CO_3 solutions in the 25 – 700 °C temperature range.

Temperature (°C)	K_2CO_3 concentration (mol/kg)	CO_3^{2-} Raman band [a]		H_2O Raman band		$R_{intensity}$	R_{area}
		Intensity	Area	Intensity	Area		
24	1.03	11764	1.621E+05	50969	1.476E+07	0.231	0.011
24	2.14	21213	3.118E+05	46689	1.462E+07	0.454	0.021
24	3.39	34745	4.894E+05	45436	1.485E+07	0.765	0.033
24	4.84	46355	6.574E+05	42423	1.466E+07	1.093	0.045
24	6.50	53390	8.021E+05	36443	1.381E+07	1.465	0.058
25	1.29	2628	3.881E+04	9041	2.504E+06	0.291	0.015
100	1.29	2291	4.605E+04	7695	2.237E+06	0.298	0.021
200	1.29	1891	5.393E+04	8158	2.187E+06	0.232	0.025
300	1.29	1235	2.207E+04	7096	1.919E+06	0.174	0.012
400	1.29	661	1.281E+04	6331	1.582E+06	0.104	0.008
25	2.36	4604	6.796E+04	8635	2.480E+06	0.533	0.027
100	2.36	4270	6.485E+04	7825	2.206E+06	0.546	0.029
200	2.36	3531	6.346E+04	7415	2.014E+06	0.476	0.032
300	2.36	2771	4.885E+04	6828	1.827E+06	0.406	0.027
400	2.36	1836	5.149E+04	6278	1.634E+06	0.292	0.032
500	2.36	1163	3.769E+04	6152	1.549E+06	0.189	0.024
600	2.36	464	6.750E+03	6568	1.486E+06	0.071	0.005
25	3.52	6171	8.769E+04	7749	2.430E+06	0.796	0.036
100	3.52	5880	9.506E+04	7316	2.190E+06	0.804	0.043
200	3.52	5131	9.948E+04	7295	2.120E+06	0.703	0.047
300	3.52	4482	8.555E+04	6898	2.133E+06	0.650	0.040
400	3.52	3586	8.842E+04	6869	1.961E+06	0.522	0.045
500	3.52	2219	7.679E+04	6176	1.754E+06	0.359	0.044
600	3.52	1482	5.618E+04	5865	1.557E+06	0.253	0.036
700	3.52	598	1.107E+04	5949	1.497E+06	0.101	0.007
25	5.14	10550	1.553E+05	9075	3.145E+06	1.163	0.049

100	5.14	10009	1.552E+05	9046	2.950E+06	1.107	0.053
200	5.14	8531	1.453E+05	7999	2.441E+06	1.066	0.060
300	5.14	7277	1.443E+05	7460	2.255E+06	0.975	0.064
400	5.14	5889	1.251E+05	7118	2.059E+06	0.827	0.061
500	5.14	4794	1.087E+05	7008	1.879E+06	0.684	0.058
600	5.14	3566	7.748E+04	7209	1.946E+06	0.495	0.040
700	5.14	2315	7.523E+04	6717	1.657E+06	0.345	0.045
25	6.63	11744	1.812E+05	7828	2.718E+06	1.500	0.067
100	6.63	12184	1.984E+05	8378	2.887E+06	1.454	0.069
200	6.63	10913	1.965E+05	8174	2.837E+06	1.335	0.069
300	6.63	9470	1.904E+05	7690	2.393E+06	1.231	0.080
400	6.63	7578	1.697E+05	6986	2.034E+06	1.085	0.083
500	6.63	6400	1.539E+05	6922	2.001E+06	0.925	0.077
600	6.63	5131	1.259E+05	6693	1.881E+06	0.767	0.067
700	6.63	3186	9.709E+04	6186	1.597E+06	0.515	0.061
26	8.12 [b]	12492	1.994E+05	6873	2.659E+06	1.818	0.075
100	9.86 [c]	11071	1.681E+05	5287	1.758E+06	2.094	0.096
200	9.86	8310	1.637E+05	4269	1.569E+06	1.946	0.104
300	9.86	8734	1.581E+05	4756	1.412E+06	1.836	0.112
400	9.86	7118	1.549E+05	4323	1.601E+06	1.647	0.097
500	9.86	5985	1.250E+05	4190	1.262E+06	1.428	0.099
600	9.86	4383	1.030E+05	3492	1.024E+06	1.255	0.101
700	9.86	3714	8.786E+04	3581	1.171E+06	1.037	0.075
100	11.28 [d]	15079	2.524E+05	6359	2.271E+06	2.371	0.111
200	12.72 [e]	10420	1.675E+05	4154	1.397E+06	2.508	0.120
300	12.72	12199	2.228E+05	5313	1.799E+06	2.296	0.124
400	12.72	11553	2.341E+05	5503	1.790E+06	2.099	0.131
500	12.72	9737	1.898E+05	5150	1.532E+06	1.891	0.124
600	12.72	7764	1.632E+05	4672	1.376E+06	1.662	0.119
700	12.72	6135	1.268E+05	4330	1.122E+06	1.417	0.113
200	18.80 [f]	11415	1.606E+05	3244	1.075E+06	3.519	0.149
300	18.80	11053	2.138E+05	3366	1.396E+06	3.283	0.153

400	18.80	9192	1.718E+05	3002	1.136E+06	3.062	0.151
500	18.80	8059	1.884E+05	2818	1.290E+06	2.859	0.146
600	18.80	7659	1.761E+05	2964	1.230E+06	2.584	0.143
700	18.80	6040	1.626E+05	2636	1.183E+06	2.292	0.137

Note: [a] – CO_3^{2-} Raman peak intensity and area are integrated values of the CO_3^{2-} and calibrated HCO_3^- bands, same for the data in Tables S4 and S5;

[b – f] – CO_3^{2-} concentrations were calculated based on the solubility of K_2CO_3 (*34*) at 26 °C, 74 °C, 100 °C, 122 °C and 189 °C, respectively.

(3) Effects from alkali cations on the Raman quantification results for CO_3^{2-} concentration

In principle, the substitution for K^+ by Na^+ or other alkali cations in aqueous solutions has little influence on the Raman peak intensities and areas of isolated CO_3^{2-} anions. This has been confirmed by the results from Sun and Qin (*70*), where the CO_3^{2-}/H_2O Raman peak intensity ratios measured with Na_2CO_3 and K_2CO_3 solutions under ambient temperature increased along the same curve with increasing CO_3^{2-} concentration. In the present study, to validate the quantification for CO_3^{2-} concentration in high *P*-*T* Na_2CO_3 and other alkaline carbonate solutions using $R_{intensity}$ and Eq. 2, two Na_2CO_3 solutions (1.45 and 3.00 mol/kg) and 3 Cs_2CO_3 solutions (1.00, 3.00 and 4.50 mol/kg) were prepared. According to the Raman quantification results, the CO_3^{2-} concentration in the 1.45 and 3.00 mol/kg Na_2CO_3 solutions were 1.37 – 1.53 mol/kg between 24 and 400 °C (CO_3^{2-} Raman peak vanished above 400 °C) and 3.14 – 3.32 mol/kg between 26 and 700 °C temperature range, respectively (Table S4). Those of the 1.00, 3.00 and 4.50 mol/kg Cs_2CO_3 solutions were 1.03 –1.22 mol/kg between 22 and 400 °C (CO_3^{2-} Raman peak vanished above 400 °C), 3.06 – 3.14 mol/kg between 25 and 600 °C, and 4.56 – 4.66 mol/kg between 25 and 700 °C, respectively (Table S5). All within the estimated errors of ±0.3 mol/kg.

Nevertheless, it is important to note that the Raman scattering coefficients of water and salts are highly dependent upon the Raman instrument settings and wavelength of the laser excitation (*67*, *71*, *72*). This results in the variation in $R_{intensity}$ with temperature and CO_3^{2-} concentration varying remarkably among the Raman spectra collected using a different spectrometer or using the same spectrometer but with different settings (*66*). In this regard, reliable quantification for CO_3^{2-} concentration in ambient and high *P*-*T* solutions can only be achieved after the variation in $R_{intensity}$ with temperature and CO_3^{2-} concentration had been calibrated properly.

Table S4 Raman quantification results for CO_3^{2-} concentration in 1.45 and 3.00 mol/kg Na_2CO_3 solutions using $R_{intensity}$ and Eq. 2.

Temperature (°C)	Na_2CO_3 concentration (mol/kg)	CO_3^{2-} Raman band		H_2O Raman band		$R_{intensity}$	CO_3^{2-} concentration (mol/kg)
		Intensity	Area	Intensity	Area		
24	1.45	4351	6.992E+04	12426	3.470E+06	0.350	1.53
100	1.45	4720	7.036E+04	13683	4.220E+06	0.345	1.51
200	1.45	4054	7.501E+04	14290	3.911E+06	0.284	1.37
300	1.45	3338	7.951E+04	14933	3.453E+06	0.224	1.39
400	1.45	1826	3.551E+04	13228	2.643E+06	0.138	1.41
26	3.00	9284	1.404E+05	12800	4.414E+06	0.725	3.15
100	3.00	9202	1.496E+05	13051	4.324E+06	0.705	3.17
200	3.00	8345	1.510E+05	12964	3.694E+06	0.644	3.16
300	3.00	6198	1.356E+05	10977	3.321E+06	0.565	3.21
400	3.00	4661	1.076E+05	10568	2.737E+06	0.441	3.14
500	3.00	3855	8.230E+04	12099	2.483E+06	0.319	3.18
600	3.00	2063	4.861E+04	11454	2.149E+06	0.180	3.21
700	3.00	482	1.254E+04	12682	2.412E+06	0.038	3.32

Table S5 Raman quantification results for CO_3^{2-} concentration in 1.0, 3.0 and 4.5 mol/kg Cs_2CO_3 solutions using $R_{\text{intensity}}$ and Eq. 2.

Temperature (°C)	Cs_2CO_3 concentration (mol/kg)	CO_3^{2-} Raman band		H_2O Raman band		$R_{\text{intensity}}$	CO_3^{2-} concentration (mol/kg)
		Intensity	Area	Intensity	Area		
22	1.00	3949	7.075E+04	15357	4.059E+06	0.257	1.14
100	1.00	3399	6.136E+04	14021	3.884E+06	0.242	1.03
200	1.00	2702	6.523E+04	12190	3.074E+06	0.222	1.04
300	1.00	2085	5.183E+04	12129	2.943E+06	0.172	1.09
400	1.00	1227	3.771E+04	11274	2.681E+06	0.109	1.22
25	3.00	10408	1.710E+05	14748	4.439E+06	0.706	3.06
100	3.00	8746	1.496E+05	12635	3.870E+06	0.692	3.07
200	3.00	6954	1.233E+05	10885	3.084E+06	0.639	3.07
300	3.00	5447	1.221E+05	9713	2.843E+06	0.561	3.11
400	3.00	4596	1.425E+05	10158	2.947E+06	0.452	3.11
500	3.00	3114	9.551E+04	9499	1.996E+06	0.328	3.14
600	3.00	1735	5.549E+04	9719	1.773E+06	0.178	3.14
25	4.50	14752	2.307E+05	14052	4.855E+06	1.050	4.58
100	4.50	12832	2.229E+05	12634	4.120E+06	1.016	4.58
200	4.50	11579	2.456E+05	12199	4.091E+06	0.949	4.62
300	4.50	9753	2.177E+05	11563	3.649E+06	0.843	4.60
400	4.50	8790	2.070E+05	12345	3.246E+06	0.712	4.56
500	4.50	6366	1.646E+05	11147	2.849E+06	0.571	4.58
600	4.50	4732	1.454E+05	11310	2.422E+06	0.418	4.62
700	4.50	3112	5.965E+04	12515	1.692E+06	0.249	4.66

REFERENCES AND NOTES

1. G. M. Yaxley, M. Anenburg, S. Tappe, S. Decree, T. Guzmics, Carbonatites: Classification, sources, evolution, and emplacement. *Annu. Rev. Earth Planet. Sci.* **50**, 261–293 (2022).

2. W. Chen, V. S. Kamenetsky, A. Simonetti, Evidence for the alkaline nature of parental carbonatite melts at Oka complex in Canada. *Nat. Commun.* **4**, 2687 (2013).

3. P. L. Verplanck, A. N. Mariano, A. Mariano Jr., Rare earth element ore geology of carbonatites, in *Rare Earth and Critical Elements in Ore Deposits*, P. L. Verplanck, M. W. Hitzman, Eds. (Society of Economic Geologists, 2016), vol. 18, pp. 5–32.

4. A. R. Woolley, D. R. C. Kempe, Carbonatites: Nomenclature, average chemical compositions, and element distribution, in *Carbonatites: Genesis and Evolution,* K. Bell, Ed. (Unwin Hyman, 1989), chap. 1, pp. 1–14.

5. E. R. Humphreys-Williams, S. Zahirovic, Carbonatites and global tectonics. *Elements* **17**, 339–344 (2021).

6. A. N. Zaitsev, J. Keller, Mineralogical and chemical transformation of Oldoinyo Lengai natrocarbonatites, Tanzania. *Lithos* **91**, 191–207 (2006).

7. J. Berndt, S. Klemme, Origin of carbonatites—Liquid immiscibility caught in the act. *Nat. Commun.* **13**, 2892 (2022).

8. I. R. Prokopyev, A. S. Borisenko, A. A. Borovikov, G. G. Pavlova, Origin of REE-rich ferrocarbonatites in southern Siberia (Russia): Implications based on melt and fluid inclusions. *Mineral. Petrol.* **110**, 845–859 (2016).

9. H. A. L. Elliott, F. Wall, A. R. Chakhmouradian, P. R. Siegfried, S. Dahlgren, S. Weatherley, A. A. Finch, M. A. W. Marks, E. Dowman, E. Deady, Fenites associated with carbonatite complexes: A review. *Ore Geol. Rev.* **93**, 38–59 (2018).

10. B. F. Walter, R. J. Giebel, M. Steele-MacInnis, M. A. W. Marks, J. Kolb, G. Markl, Fluids associated with carbonatitic magmatism: A critical review and implications for carbonatite magma ascent. *Earth Sci. Rev.* **215**, 103509 (2021).

11. M. Anenburg, A. M. John, C. Frigo, F. Wall, Rare earth element mobility in and around carbonatites controlled by sodium, potassium, and silica. *Sci. Adv.* **6**, eabb6570 (2020).

12. D. Weidendorfer, M. W. Schmidt, H. B. Mattsson, A common origin of carbonatite magmas. *Geology* **45**, 507–510 (2017).

13. V. Mollé, F. Gaillard, Z. Nabyl, J. Tuduri, I. D. Carlo, S. Erdmann, Crystallisation sequence of a REE-rich carbonate melt: An experimental approach. *C. R. Geosci.* **353**, 217–231 (2021).

14. M. Anenburg, S. Broom-Fendley, W. Chen, Formation of rare earth deposits in carbonatites. *Elements* **17**, 327–332 (2021).

15. D. A. Chebotarev, I. V. Veksler, C. Wohlgemuth-Ueberwasser, A. G. Doroshkevich, M. Koch-Müller, Experimental study of trace element distribution between calcite, fluorite and carbonatitic melt in the system $CaCO_3 + CaF_2 + Na_2CO_3 \pm Ca_3(PO_4)_2$ at 100 MPa. *Contrib. Mineral. Petrol.* **174**, 4 (2019).

16. A. G. Doroshkevich, I. V. Veksler, R. Klemd, E. A. Khromova, I. A. Izbrodin, Trace-element composition of minerals and rocks in the Belaya Zima carbonatite complex (Russia): Implications for the mechanisms of magma evolution and carbonatite formation. *Lithos* **284-285**, 91–108 (2017).

17. D. Ionov, R. E. Harmer, Trace element distribution in calcite–dolomite carbonatites from Spitskop: Inferences for differentiation of carbonatite magmas and the origin of carbonates in mantle xenoliths. *Earth Planet. Sci. Lett.* **198**, 495–510 (2002).

18. Y. Xie, Z. Hou, S. Yin, S. C. Dominy, J. Xu, S. Tian, W. Xu, Continuous carbonatitic melt–fluid evolution of a REE mineralization system: Evidence from inclusions in the Maoniuping REE Deposit, Western Sichuan, China. *Ore Geol. Rev.* **36**, 90–105 (2009).

19. J. Gittins, M. F. Beckett, B. C. Jago, Composition of the fluid phase accompanying carbonatite magma; a critical examination. *Am. Mineral.* **75**, 1106–1109 (1990).

20. R. H. Mitchell, Carbonate-carbonate immiscibility, neighborite and potassium iron sulphide in Oldoinyo Lengai natrocarbonatite. *Mineral. Mag.* **61**, 779–789 (1997).

21. W. Song, C. Xu, I. V. Veksler, J. Kynicky, Experimental study of REE, Ba, Sr, Mo and W partitioning between carbonatitic melt and aqueous fluid with implications for rare metal mineralization. *Contrib. Mineral. Petrol.* **171**, 1 (2015).

22. I. V. Veksler, H. Keppler, Partitioning of Mg, Ca, and Na between carbonatite melt and hydrous fluid at 0.1–0.2 GPa. *Contrib. Mineral. Petrol.* **138**, 27–34 (2000).

23. A. G. Doroshkevich, S. G. Viladkar, G. S. Ripp, M. V. Burtseva, Hydrothermal REE mineralization in the Amba Dongar carbonatite complex, Gujarat, India. *Can. Mineral.* **47**, 1105–1116 (2009).

24. R. J. Giebel, C. D. K. Gauert, M. A. W. Marks, G. Costin, G. Markl, Multi-stage formation of REE minerals in the Palabora Carbonatite Complex, South Africa. *Am. Mineral.* **102**, 1218–1233 (2017).

25. B. Bühn, A. H. Rankin, M. Radtke, M. Haller, A. Knöchel, Burbankite, a (Sr, REE, Na, Ca)-carbonate in fluid inclusions from carbonatite-derived fluids; identification and characterization using laser Raman spectroscopy, SEM-EDX, and synchrotron micro-XRF analysis. *Am. Mineral.* **84**, 1117–1125 (1999).

26. A. Costanzo, K. R. Moore, F. Wall, M. Feely, Fluid inclusions in apatite from Jacupiranga calcite carbonatites: Evidence for a fluid-stratified carbonatite magma chamber. *Lithos* **91**, 208–228 (2006).

27. A. R. Chakhmouradian, S. Dahlgren, Primary inclusions of burbankite in carbonatites from the Fen complex, southern Norway. *Mineral. Petrol.* **115**, 161–171 (2021).

28. B. Bühn, A. H. Rankin, Composition of natural, volatile-rich Na–Ca–REE−Sr carbonatitic fluids trapped in fluid inclusions. *Geochim. Cosmochim. Acta* **63**, 3781–3797 (1999).

29. B. Bühn, A. H. Rankin, J. Schneider, P. Dulski, The nature of orthomagmatic, carbonatitic fluids precipitating REE,Sr-rich fluorite: Fluid-inclusion evidence from the Okorusu fluorite deposit, Namibia. *Chem. Geol.* **186**, 75–98 (2002).

30. A. N. Zaitsev, A. Demény, S. Sindern, F. Wall, Burbankite group minerals and their alteration in rare earth carbonatites—Source of elements and fluids (evidence from C–O and Sr–Nd isotopic data). *Lithos* **62**, 15–33 (2002).

31. M. Hutchinson, P. Slezak, R. Wendlandt, M. Hitzman, Rare rarth element enrichment in the weathering profile of the Bull Hill carbonatite at Bear Lodge, Wyoming, USA. *Econ. Geol.* **117**, 813–831 (2022).

32. M. A. Sitnikova, V. Do Cabo, F. Wall, S. Goldmann, Burbankite and pseudomorphs from the Main Intrusion calcite carbonatite, Lofdal, Namibia: Association, mineral composition, Raman spectroscopy. *Mineral. Mag.* **85**, 496–513 (2021).

33. S. B. Castor, The mountain pass rare-earth carbonatite and associated ultrapotassic rocks, California. *Can. Mineral.* **46**, 779–806 (2008).

34. H. Schultz, G. Bauer, E. Schachl, F. Hagedorn, P. Schmittinger, Potassium compounds, in *Ullmann's Encyclopedia of Industrial Chemistry,* Wiley-VCH, Ed. (Wiley-VCH, ed 7, 2011), vol. 29, pp. 1–65.

35. V. M. Valyashko, Phase equilibria of water-salt systems at high temperatures and pressures, in *Aqueous Systems at Elevated Temperatures and Pressures,* D. A. Palmer, R. Fernández-Prini, A. H. Harvey, Eds. (Academic Press, 2004), pp. 597–641.

36. M. S. Khan, S. N. Rogak, Solubility of Na_2SO_4, Na_2CO_3 and their mixture in supercritical water. *J. Supercrit. Fluid.* **30**, 359–373 (2004).

37. G. Lemoine, H.-A. Turc, A. Leybros, J.-C. Ruiz, Y. Sommer de Gélicourt, H. Muhr, Na_2CO_3 and K_3PO_4 solubility measurements at 30 MPa in near-critical and supercritical water using conductimetry and high pressure calorimetry. *J. Supercrit. Fluid.* **130**, 91–96 (2017).

38. A. Apelblat, E. Korin, E. Manzurola, Solubilities and vapour pressures of saturated aqueous solutions of sodium peroxydisulfate and potassium peroxydisulfate. *J. Chem. Thermodyn.* **33**, 61–69 (2001).

39. W. F. Waldeck, G. Lynn, A. E. Hill, Aqueous solubility of salts at high temperatures. I. Solubility of sodium carbonate from 50 to 348 °C. *J. Am. Chem. Soc.* **54**, 928–936 (1932).

40. Z. A. Kotel'nikova, A. R. Kotel'nikov, Na_2CO_3-bearing fluids: Experimental study at 700°C and under 1, 2, and 3 kbar pressure using synthetic fluid inclusions in quartz. *Geol. Ore Deposits* **53**, 156–170 (2011).

41. I. V. Veksler, Liquid immiscibility and its role at the magmatic–hydrothermal transition: A summary of experimental studies. *Chem. Geol.* **210**, 7–31 (2004).

42. X. Zheng, Y. Liu, L. Zhang, The role of sulfate-, alkali-, and halogen-rich fluids in mobilization and mineralization of rare earth elements: Insights from bulk fluid compositions in the Mianning–Dechang carbonatite-related REE belt, southwestern China. *Lithos* **386-387**, 106008 (2021).

43. A. K. van Groos, High-pressure DTA study of the upper three-phase region in the system Na_2CO_3-H_2O. *Am. Mineral.* **75**, 667–675 (1990).

44. V. Hurai, M. Blažeková, M. Huraiová, P. R. Siegfried, M. Slobodník, P. Konečný, Thermobarometric and geochronologic constraints on the emplacement of the Neoproterozoic Evate carbonatite during exhumation of the Monapo granulite complex, Mozambique. *Lithos* **380-381**, 105883 (2021).

45. Y. Liu, Z. Hou, A synthesis of mineralization styles with an integrated genetic model of carbonatite-syenite-hosted REE deposits in the Cenozoic Mianning-Dechang REE metallogenic belt, the eastern Tibetan Plateau, southwestern China. *J. Asian Earth Sci.* **137**, 35–79 (2017).

46. X. Yuan, R. A. Mayanovic, An empirical study on Raman peak fitting and its application to Raman quantitative research. *Appl. Spectrosc.* **71**, 2325–2338 (2017).

47. M. I. Ravich, F. E. Borovaya, The solubility of sodium carbonate in water at elevated temperatures and pressures. *Dokl. Akad. Nauk SSSR* **156**, 894–897 (1964).

48. D. Mantegazzi, "PVTx properties of saline aqueous fluids at high P-T conditions from acoustic velocity measurements using Brillouin scattering spectroscopy", thesis, ETH (2012).

49. B. F. Walter, M. Steele-MacInnis, R. J. Giebel, M. A. W. Marks, G. Markl, Complex carbonate-sulfate brines in fluid inclusions from carbonatites: Estimating compositions in the system H_2O-Na-K-CO_3-SO_4-Cl. *Geochim. Cosmochim. Acta* **277**, 224–242 (2020).

50. A. E. Williams-Jones, D. A. S. Palmer, The evolution of aqueous–carbonic fluids in the Amba Dongar carbonatite, India: Implications for fenitisation. *Chem. Geol.* **185**, 283–301 (2002).

51. M. Poutiainen, Fluids in the Siilinjärvi carbonatite complex, eastern Finland: Fluid inclusion evidence for the formation conditions of zircon and apatite. *B. Geol. Soc. Finland* **67**, 3–18 (1995).

52. S. Broom-Fendley, A. E. Brady, F. Wall, G. Gunn, W. Dawes, REE minerals at the Songwe Hill carbonatite, Malawi: HREE-enrichment in late-stage apatite. *Ore Geol. Rev.* **81**, 23–41 (2017).

53. M.-L. Frezzotti, J. L. R. Touret, CO_2, carbonate-rich melts, and brines in the mantle. *Geosci. Front.* **5**, 697–710 (2014).

54. E. Kozlov, E. Fomina, M. Sidorov, V. Shilovskikh, V. Bocharov, A. Chernyavsky, M. Huber, The Petyayan-Vara carbonatite-hosted rare earth deposit (Vuoriyarvi, NW Russia): Mineralogy and geochemistry. *Minerals* **10**, 73 (2020).

55. I. Prokopyev, E. Kozlov, E. Fomina, A. Doroshkevich, M. Dyomkin, Mineralogy and fluid regime of formation of the REE-late-stage hydrothermal mineralization of Petyayan-Vara carbonatites (Vuoriyarvi, Kola Region, NW Russia). *Minerals* **10**, 405 (2020).

56. V. V. Sharygin, L. M. Zhitova, E. N. Nigmatulina, Fairchildite $K_2Ca(CO_3)_2$ in phoscorites from Phalaborwa, South Africa: The first occurrence in alkaline carbonatite complexes. *Russ. Geol. Geophys.* **52**, 208–219 (2011).

57. H. O'Brien, E. Heilimo, P. Heino, The archean Siilinjärvi carbonatite complex, in *Mineral Deposits of Finland,* W. D. Maier, R. Lahtinen, H. O'Brien, Eds. (Elsevier, 2015), pp. 327–343.

58. W. A. Bassett, A. H. Shen, M. Bucknum, I. M. Chou, A new diamond anvil cell for hydrothermal studies to 2.5 GPa and from −190 to 1200 °C. *Rev. Sci. Instrum.* **64**, 2340–2345 (1993).

59. C. Schmidt, I. M. Chou, The hydrothermal diamond anvil Cell (HDAC) for raman spectroscopic studies of geologic fluids at high pressures and temperatures, in *Raman Spectroscopy Applied to Earth Sciences and Cultural Heritage,* J. Dubessy, M.-C. Caumon, F. R. Pérez, Eds. (European Mineralogical Union, 2012), vol. 12, chap. 7, pp. 247–276.

60. C. Schmidt, M. Steelemacinnis, A. Watenphul, M. Wilke, Calibration of zircon as a Raman spectroscopic pressure sensor to high temperatures and application to water-silicate melt systems. *Am. Mineral.* **98**, 643–650 (2013).

61. C. Schmidt, M. A. Ziemann, In-situ Raman spectroscopy of quartz: A pressure sensor for hydrothermal diamond-anvil cell experiments at elevated temperatures. *Am. Mineral.* **85**, 1725–1734 (2000).

62. X. Yuan, R. A. Mayanovic, H. Zheng, Determination of pressure from measured Raman frequency shifts of anhydrite and its application in fluid inclusions and HDAC experiments. *Geochim. Cosmochim. Acta* **194**, 253–265 (2016).

63. R. J. Bodnar, Introduction to aqueous-electrolyte fluid inclusions, in *Fluid Inclusions, Analysis and Interpretation,* I. Samson, A. Anderson, D. Marshall, Eds. (Mineralogical Association of Canada, Short Course Series, 2003), vol. 32, chap. 3, pp. 9–53.

64. X. Yuan, X. Xiong, G. Zhang, R. A. Mayanovic, Application of calcite, Mg-calcite, and dolomite as Raman pressure sensors for high-pressure, high-temperature studies. *J. Raman Spectrosc.* **51**, 1248–1259 (2020).

65. C. Schmidt, Raman spectroscopic determination of carbon speciation and quartz solubility in H_2O + Na_2 CO_3 and H_2O + $NaHCO_3$ fluids to 600 °C and 1.53 GPa. *Geochim. Cosmochim. Acta* **145**, 281–296 (2014).

66. Y. Ma, W. Yan, Q. Sun, X. Liu, Raman and infrared spectroscopic quantification of the carbonate concentration in K_2CO_3 aqueous solutions with water as an internal standard. *Geosci. Front.* **12**, 1018–1030 (2021).

67. W. W. Rudolph, G. Irmer, E. Königsberger, Speciation studies in aqueous HCO_3^-–CO_3^{2-} solutions. A combined Raman spectroscopic and thermodynamic study. *Dalton Trans.* 900–908 (2008).

68. J. P. Hershey, S. Sotolongo, F. J. Millero, Densities and compressibilities of aqueous sodium carbonate and bicarbonate from 0 to 45°C. *J. Solution Chem.* **12**, 233–254 (1983).

69. A. V. Sharygin, R. H. Wood, Densities of aqueous solutions of sodium carbonate and sodium bicarbonate at temperatures from (298 to 623) K and pressures to 28 MPa. *J. Chem. Thermodyn.* **30**, 1555–1570 (1998).

70. Q. Sun, C. Qin, Raman OH stretching band of water as an internal standard to determine carbonate concentrations. *Chem. Geol.* **283**, 274–278 (2011).

71. C. Schmidt, T. M. Seward, Raman spectroscopic quantification of sulfur species in aqueous fluids: Ratios of relative molar scattering factors of Raman bands of H_2S, HS^-, SO_2, HSO_4^-, SO_4^{2-}, $S_2O_3^{2-}$, S^{3-} and H_2O at ambient conditions and information on changes with pressure and temperature. *Chem. Geol.* **467**, 64–75 (2017).

72. J. D. Frantz, Raman spectra of potassium carbonate and bicarbonate aqueous fluids at elevated temperatures and pressures: Comparison with theoretical simulations. *Chem. Geol.* **152**, 211–225 (1998).
